# AgNPs Change Microbial Community Structures of Wastewater

**DOI:** 10.3389/fmicb.2018.03211

**Published:** 2019-01-08

**Authors:** Yuting Guo, Nicolas Cichocki, Florian Schattenberg, Robert Geffers, Hauke Harms, Susann Müller

**Affiliations:** ^1^Department of Environmental Microbiology, Helmholtz Centre for Environmental Research, Leipzig, Germany; ^2^Research Group Genome Analysis, Helmholtz Centre for Infection Research, Braunschweig, Germany

**Keywords:** silver nanoparticles, wastewater microbial community, microbial diversity, single cell analysis, microbial ecotoxicology, silver toxicity

## Abstract

Due to their strong antimicrobial activity, silver nanoparticles (AgNPs) are massively produced, applied, consumed and, as a negative consequence, released into wastewater treatment plants. Most AgNPs are assumed to be bound by sludge, and thus bear potential risk for microbial performance and stability. In this lab-scale study, flow cytometry as a high-throughput method and 16S rRNA gene amplicon Illumina MiSeq sequencing were used to track microbial community structure changes when being exposed to AgNPs. Both methods allowed deeper investigation of the toxic impact of chemicals on microbial communities than classical EC_50_ determination. In addition, ecological metrics were used to quantify microbial community variations depending on AgNP types (10 and 30 nm) and concentrations. Only low changes in α- and intra-community β-diversity values were found both in successive negative and positive control batches and batches that were run with AgNPs below the EC_50_ value. Instead, AgNPs at EC_50_ concentrations caused upcoming of certain and disappearance of formerly dominant subcommunities. *Flavobacteriia* were among those that almost disappeared, while phylotypes affiliated with *Gammaproteobacteria* (3.6-fold) and *Bacilli* (8.4-fold) increased in cell abundance in comparison to the negative control. Thus, silver amounts at the EC_50_ value affected community structure suggesting a potential negative impact on functions in wastewater treatment systems.

## Introduction

Silver nanoparticles (AgNPs) are incorporated into various consumer products due to their efficient antimicrobial activity (Chen and Schluesener, 2008; Rai et al., 2009; Wijnhoven et al., 2009; Marambio-Jones and Hoek, 2010). The global production of AgNPs has been estimated to reach ~800 tons by the year 2025 (Pulit-Prociak and Banach, 2016). Along with the increasing production, the application and consumption of AgNP-containing products are known to result in AgNP-release into wastewater treatment systems (Benn and Westerhoff, 2008; Voelker et al., 2015) with up to 50 g/d being detected in the influent of full-scale municipal wastewater treatment plants (WWTPs) (Li et al., 2016). The impact of the antimicrobial activities of silver on the structure and function of microbial wastewater communities is under-investigated. This study wants to test if AgNPs act on persistence of whole microbial communities, or selectively on certain cell types thereby possibly influencing the water purification process.

It has been shown that most of the silver in the WWTPs will be captured and settled in the sludge (Hendren et al., 2013; Barton et al., 2015; Oh et al., 2015). Field analyses of WWTPs estimated that up to 95% of the silver was eliminated from the wastewater by binding to sludge; and silver concentrations in sludge between 1 and 6 mg/kg can be expected (Gottschalk et al., 2009; Li et al., 2016). Batch studies confirmed also for AgNPs (20 and 60 nm) at concentrations of 0.5–10 mg/L more than 90% binding to sludge after a contact time of 6 h (Tiede et al., 2010). Generally, sludge from WWTPs is either incinerated, applied in agriculture or deposited in landfills. According to the European Commission^1^, more than 70% of the total volume of treated sludge is still used in agriculture as fertilizer e.g., in Portugal, Ireland, United Kingdom, and Albania. Since the sludge is recirculated several times within a WWTP before removal, with retention times ranging from 5 to 27 d (Choubert et al., 2011; Jelic et al., 2011) AgNPs embedded within sludge may represent a considerable potential hazard for the activity of key species both within the several operational steps in a WWTP and, later, in agriculture due to their strong antimicrobial activity.

The negative effects of AgNPs on microbial organisms attract increasing attention. On the lab-scale, pure-culture studies mainly on *Pseudomonas, Escherichia, Staphylococcus*, and *Bacillus* strains were performed to understand the action of silver ions and AgNPs on bacteria (Kim et al., 2007; Yoon et al., 2007; Jin et al., 2010; Suresh et al., 2010; Guo et al., 2017a,b). Toxic effects of AgNPs have been related to cell membrane damage, generation of reactive oxygen species, disruption of the function of biomolecules such as proteins, enzymes, plasmids, and DNA (Reidy et al., 2013; von Moos and Slaveykova, 2014). Silver ions are now commonly believed to be the principle toxic agent over AgNPs (Lok et al., 2007; Xiu et al., 2012; Bondarenko et al., 2013; Visnapuu et al., 2013) which severely affect bacterial growth and function. But AgNP-aggregates enhance this toxicity further since they act as source for a steady release of silver ions into the environment (Guo et al., 2017b). The stability and dissolution of AgNP-aggregates and the resulting toxicity have been reported to be strongly dependent on environmental conditions, such as the ionic strength, the sorption of organic and inorganic species and, therefore, on changes in oxidation and aggregation states (Liu and Hurt, 2010; Xiu et al., 2011; Domingo et al., 2019).

Apart from pure culture studies, microbial communities in complex AgNP-affected environments such as WWTPs were also investigated. In fact it has been reported that microbial communities can serve as indicators for chemical pollution as they are ubiquitously distributed and rapidly respond to environmental disturbances by changes in taxonomic and functional biodiversity (Pesce et al., 2017; Storck et al., 2018). In AgNP-affected environments sequencing approaches are commonly used to resolve microbial community structure changes. But they are impractical for rapid and cheap enumeration of time series as may be necessary for steadily changing environments that are found in WWTPs. Instead, flow cytometry and related bioinformatics tools have been proven as powerful means for near on-line monitoring the performance of wastewater treatment systems with high temporal resolution (Günther et al., 2012, 2016; Koch et al., 2014). As a rapid, low-cost, and on-site observation method, flow cytometry measures light scatter and fluorescence characteristics of every single cell in a community sample and allows the precise quantification of cells with similar properties (Liu et al., 2018). Therefore, in this study, flow cytometry was used to follow dynamic changes of arising and disappearing subcommunities to reveal diverging and segregated responses of microbial communities to silver stress. In parallel, a selected cell sorting of responding subcommunities and their meta-profiling of the V3-V4 region of the 16S rRNA gene was undertaken to identify phenotypes that might have been affected by AgNPs. Two sizes of 10 and 30 nm AgNPs were chosen for this study as they are among the most commonly used sizes according to the AgNP-database of the Nanotechnology Consumer Products Inventory (Vance et al., 2015). The study is expected to give indications on toxicity of AgNPs on microorganisms derived from wastewater and will discuss possible consequences of changed community structures on WWTP operation.

## Materials and Methods

### Materials

Silver nitrate (AgNO_3_) (99.9%) was purchased from Sigma-Aldrich (St. Louis, MO, USA). AgNPs were provided by nanoComposix (San Diego, CA, USA) as aqueous suspensions [citrate coated, mass concentration (Ag) 0.02 mg/mL] of the sizes 10 nm (9.4 ± 1.7 nm, AgNP-10) and 30 nm (32.7 ± 4.8 nm, AgNP-30). The nanoparticles were stored at 4°C in the dark. Prior to use, vigorous shaking for 30 s was needed to obtain a homogenous suspension. The size distribution and dissolution of the two sizes of AgNPs have been analyzed and published before (Guo et al., 2017b). The size distribution of AgNPs was determined by using the DynaPro Nano Star (WYATT Technology Europe, Dernbach, Germany). The dissolution of ions from AgNPs was determined by separating them using centrifugal ultrafiltration at 14,000 g for 20 min through an Ultra-0.5 Centrifugal Filter Unit with a nominal molecular weight limit of 3 KDa (0.1 nm pore size). The silver ion amount was measured by an Element XR Inductively Coupled Plasma Mass Spectrometer (Thermo Fisher Scientific, Langenselbold, Germany). It was found that AgNP-10 and AgNP-30 formed large aggregates in the medium with diameters of 445 ± 58 and 473 ± 65 nm, respectively; and 4.8 and 1.8% of dissolved silver ions, respectively, within a time period of 12 h.

### Experimental Setup

Wastewater was collected from an activated sludge basin at a full-scale communal WWTP in Eilenburg, Germany (51°27′39.4″N, 12°36′17.5″E, ~10,200 m^3^ wastewater per day, and water purification according to German Waste Water Ordinance–AbwV, March 2017^2^), fractionated and stored at −20°C. Medium composition is provided in Table S1. Experiments were processed in 50-mL flasks with 10-mL medium in a sequenced-batch cultivation mode. To start the experiments, wastewater fractions from a given sample were slowly defrosted. The operational steps were as follows: equal amounts of these fractions were added to each flask at an initial optical density of 0.09 (OD_600_ = 0.09, Ultrospec 1100pro, Amersham Biosciences, Buckinghamshire, UK). The wastewater microbial communities were exposed to 4 concentrations of silver (silver-confined conditions): 0.1 mg/L AgNP-10 (low concentration, LAg10) and 2.25 mg/L AgNP-10 (EC_50_Ag10), 7.13 mg/L AgNP-30 (EC_50_Ag30) and 0.25 mg/L AgNO_3_ (silver ion positive control, Pos). The concentrations of EC_50_Ag10, EC_50_Ag30 and Pos referred to determined EC_50_ values, which were the effective silver concentrations causing half maximum growth of *Escherichia coli* (Guo et al., 2017b). As a silver ion negative control (Neg) a wastewater microbial community was cultivated without any silver treatment (silver-free condition). The sampling interval was 3–4 d over a time range of 24 d. After each sampling, the wastewater microbial communities were transferred to a new flask with fresh medium, respectively, by repeating the same operational steps. The whole experiments were performed twice in independent setups (Setup 1 and Setup 2). For each treatment per setup, triplicate experiments were performed. All the data mentioned in the main text are from Setup 1, and comparable data from Setup 2 are shown in the Supplementary Materials.

### Fixation Procedures

For each sampling, 4 mL of the culture was harvested into a glass tube and centrifuged (3,200 g, 20 min, 15°C). The cell pellet was re-suspended in 4 mL of 2% paraformaldehyde (PFA) in phosphate buffered saline (PBS, compositions are given in Table S1) for 30 min at room temperature (RT), then centrifuged (3,200 g, 10 min, 15°C). After removing the supernatant the cell pellet was re-suspended in 4 mL of 70% ethanol in distilled water and stored at −20°C until staining.

### Staining Procedures

Fixed samples were diluted (0.6 mL of fixed sample+1.4 mL of PBS) and ultra-sonicated (ultrasonic bath, Merck Eurolab, Darmstadt, Germany, 35 KHz at RT) for 10 min before centrifugation (3,200 g, 10 min, 15°C). The cell pellet was washed once with 2 mL of PBS (3,200 g, 10 min, 15°C). After removing the supernatant, the cell pellet was ultra-sonicated again for 5 min. Following, the cells were adjusted to OD_700_ = 0.035 with PBS, and 2 mL of the cell suspension was taken and centrifuged (as before). Finally, the cell pellet was re-suspended in 1 mL of stock A (20 min, at RT), centrifuged (as before), re-suspended in 2 mL of stock B (overnight, dark, at RT) until flow cytometric measurement. The compositions of stock A and stock B are presented in Table S1.

### Flow Cytometry Measurement

Samples were measured with a MoFlo Legacy cell sorter (Beckman-Coulter, Brea, California, USA) which was equipped with two lasers. The 488 nm laser Genesis MX488-500 STM OPS (Coherent, Santa Clara, California, USA) at 400 mW was used for measurement of forward scatter (FSC; bandpass filter 488 ± 5 nm, neutral density filter 1.9) and side scatter (SSC; bandpass filter 488 ± 5 nm, neutral density filter 1.9, trigger signal), and the 355 nm UV laser Xcyte CY-355-150 (Lumentum, Milpitas, California, USA) at 150 mW for UV-induced fluorescence (bandpass filter 450 ± 32.5 nm). Photomultiplier tubes were purchased from Hamamatsu Photonics (Models R928 and R3896; Hamamatsu, Japan). The fluidic system was run at 56 psi (3.86 bar) with sample overpressure at maximum 0.3 psi and a 70 μm nozzle. The composition of the sheath fluid is recorded in Table S1. Daily calibration of the instrument was performed linearly with 1.0 μm blue fluorescent beads [FluoSpheres (350/440), lot-no.: F8815] and 2.0 μm yellow-green fluorescent beads [FluoSpheres (505/515), lot-no.: F8827], both from Thermo Fisher Scientific (Langenselbold, Germany). UV beads [0.5 and 1 μm, both Fluoresbrite BB Carboxylate microspheres, (360/407), lot-no.: 552744 and 499344, PolyScience, Niles, Illinois, USA] were used for calibration on the logarithmic scale and were added to each sample for measurement stability. A biological standard [*E. coli* BL21 (DE3), stationary phase of growth curve (16 h cultivation time in LB medium), fixed and stained as described before] was measured as a biological adjustment. DNA-stained samples were measured cytometrically as logarithmically scaled 2D-dot plots according to DAPI fluorescence (DNA content) and FSC (cell size related) information. For every 2D-dot plot 250,000 cells were measured.

Cell sorting was done according to a standard protocol (Koch et al., 2013). In short: each sorted sample was composed of 500,000 cells from the selected sorting gate. The sorting was done in the most accurate sort mode of the MoFlo (single and one-drop mode: highest purity 99%) at a rate not higher than 2,500 particles per second. Cells were harvested by a centrifugation step (20,000 g, 4°C, 25 min), and the pellet was frozen at −20°C for later DNA isolation and 16S rRNA gene amplicon Illumina MiSeq sequencing analysis.

### Flow Cytometric Data Analysis

All cytometrically measured samples (Setup 1: 108 samples, Setup 2: 108 samples) were used to create the gate-template (34 gates) valid for all communities analyzed in the study (Figure S1). All the raw data are available at the FlowRepository^3^. Whenever a new subcommunity became apparent, a gate was set. The final gate-template was then applied to each sample to mirror the community structure changes and extract individual gate abundances. The flowCyBar tool^4^ was used to visualize the cytometric community structure changes (Figure S2) by using gate information of the silver-free and silver-confined samples over a time range of 24 d.

### Determination of Cytometric α-Diversity and Intra-Community β-Diversity Values

The cytometric α-diversity calculated through gate-based Hill numbers (Dq = 0) (Hill, 1973) has been proven to be a reasonable and fast measure to follow the diversity's richness in a community (Günther et al., 2016; Props et al., 2016; Liu et al., 2018). Only those subcommunities were counted that pass the average cell abundance threshold of 2.9%. These subcommunities were regarded as the dominant subcommunities (Liu et al., 2018).

Cytometric intra-community β-diversity values give information on dissimilarity between sampling days and indicate time-dependent influences of the silver derivatives. Cytometric intra-community β-diversity values were calculated by counting the number of subcommunities that were unique between sampling days (Günther et al., 2016).

### Sequencing Workflow

#### DNA Extraction

The selected samples for sequencing are listed in Table S2. Unsorted samples (whole community samples) were diluted in 70 μL PBS to a final OD_700_ = 0.01. Sorted cells (500,000 cells) from each gate of interest were pelleted by centrifugation (20,000 g, 4°C, 25 min). The DNA was extracted according to the protocol of Koch et al. (2013) and stored at −20°C until library preparation.

#### Library Preparation for Illumina®

The V3-V4 region of the bacterial 16S rRNA gene region was the target of the used primers Pro341F 5′-CCTACGGGNBGCASCAG-3′ (Takahashi et al., 2014) and Pro805R 5′-GACTACNVGGGTATCTAATCC-3′ (Herlemann et al., 2011) which were synthesized by Eurofins (Eurofins Scientific, Luxembourg city, Luxembourg) as were also the 6-nt-barcoded primers for library preparation. The library was prepared by using a two-step PCR procedure. Between and after PCR steps, the amplicons were purified, their purity was tested by gel electrophoresis, and finally quantified before they were equimolarly pooled for sequencing by the Illumina MiSeq platform. Details are provided in the sequencing workflow part in the Supplementary Materials.

#### Sequencing Data Evaluation

The sequencing data evaluation was done by using the Mothur program 1.39 (Schloss et al., 2009). The chimeras were removed by using UCHIME (Edgar et al., 2011) and the OTU classification was done by using the Mothur's average neighbor clustering algorithm with a 97% sequence similarity cut-off on the SILVA database version 128 (Quast et al., 2013). The obtained data sets were normalized by a subsampling procedure to 4,483 cleaned reads per sample. All the raw data are available under the BioProject accession number: PRJNA400127. To ensure the reliability of the sequencing and evaluation procedures, two mock strains and a mock community (MBARC26), (Singer et al., 2016) were included in the analysis. The sequencing data evaluation comprised the set-up of an OTU threshold at the level of 0.71% according to recommendations of Bokulich et al. (2012). Details are in the sequencing workflow part in the Supplementary Materials.

## Results

### Fingerprinting of Silver Influenced Wastewater Microbial Community Structures

**Figure 1:**
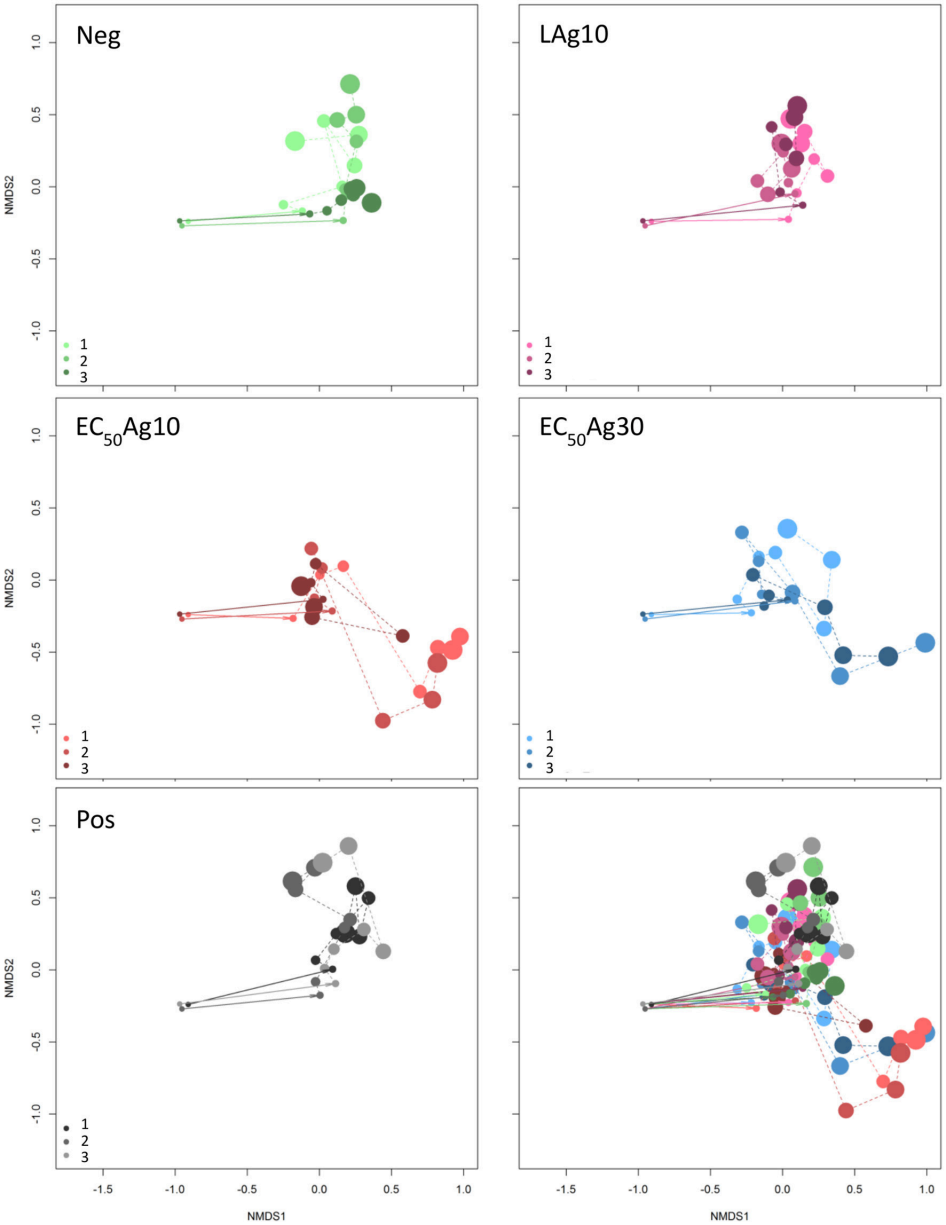
Dynamic cytometric structure changes of microbial communities. Data are shown for Setup 1 for silver ion negative control (Neg), 0.1 mg/L AgNP-10 (LAg10), 2.25 mg/L AgNP-10 (EC_50_Ag10), 7.13 mg/L AgNP-30 (EC_50_Ag30), silver ion positive control of 0.25 mg/L AgNO_3_ (Pos) (Bray-Curtis dissimilarity, stress: 0.12). Three parallel experiments were performed and shown in light, middle, and dark colors: green for Neg, pink for LAg10, red for EC_50_Ag10, blue for EC_50_Ag30, gray for Pos. The cytometric microbial community of each sampling day is shown as a dot and the dot size increases with increasing sampling time from 0 to 24 d. All five conditions are shown together in the sub-figure at the bottom-right. Comparable data for Setup 2 are shown in Figure S3.

The aim of this study was to investigate if and to what extent silver ions and nanoparticles influence microbial community structures in WWTPs, and to track the microbial community structure dynamics in response to these disturbances. AgNO_3_ and two sizes of AgNPs (10 and 30 nm) at EC_50_ concentrations and below were used as toxicants. Wastewater microbial communities were grown in a sequenced-batch cultivation mode and their growth dynamics were cytometrically analyzed by using the inherent cell information on cell light scattering and cell DNA contents. The dynamics of the five cultivated microbial communities in the two independent setups are shown in the Movies S1, S2. In the cytometric histograms, the cells with similar optical properties clustered together as a subcommunity (technically a gate). The positions and cell abundances of the subcommunities in a histogram reflected the community structures (Koch et al., 2013; Günther et al., 2016), and their relative changes were documented over 24 d by means of biostatistic tools such as non-metric multidimensional scaling (NMDS) plots based on Bray-Curtis dissimilarity (stress of 0.12). The results are shown in Figure 1. The inoculum of the WWTP derived community to the sequenced-batch cultivation conditions caused an adaptation in community structures, as was shown from the first to the second sampling point, both under silver-free and silver-confined conditions. Thereby, at low AgNP concentration treatment, the community structures showed high similarities with those of the negative control, and also with the positive control. In contrast, AgNPs (10 and 30 nm) at EC_50_ concentrations indicated different developments in community structures. Comparable results for Setup 2 are presented in Figure S3. Therefore, the EC_50_ concentrations of AgNPs (10 and 30 nm) can be expected to impact the communities to a different degree in comparison to the controls and the low AgNP concentration treatment.

**Figure 2:**
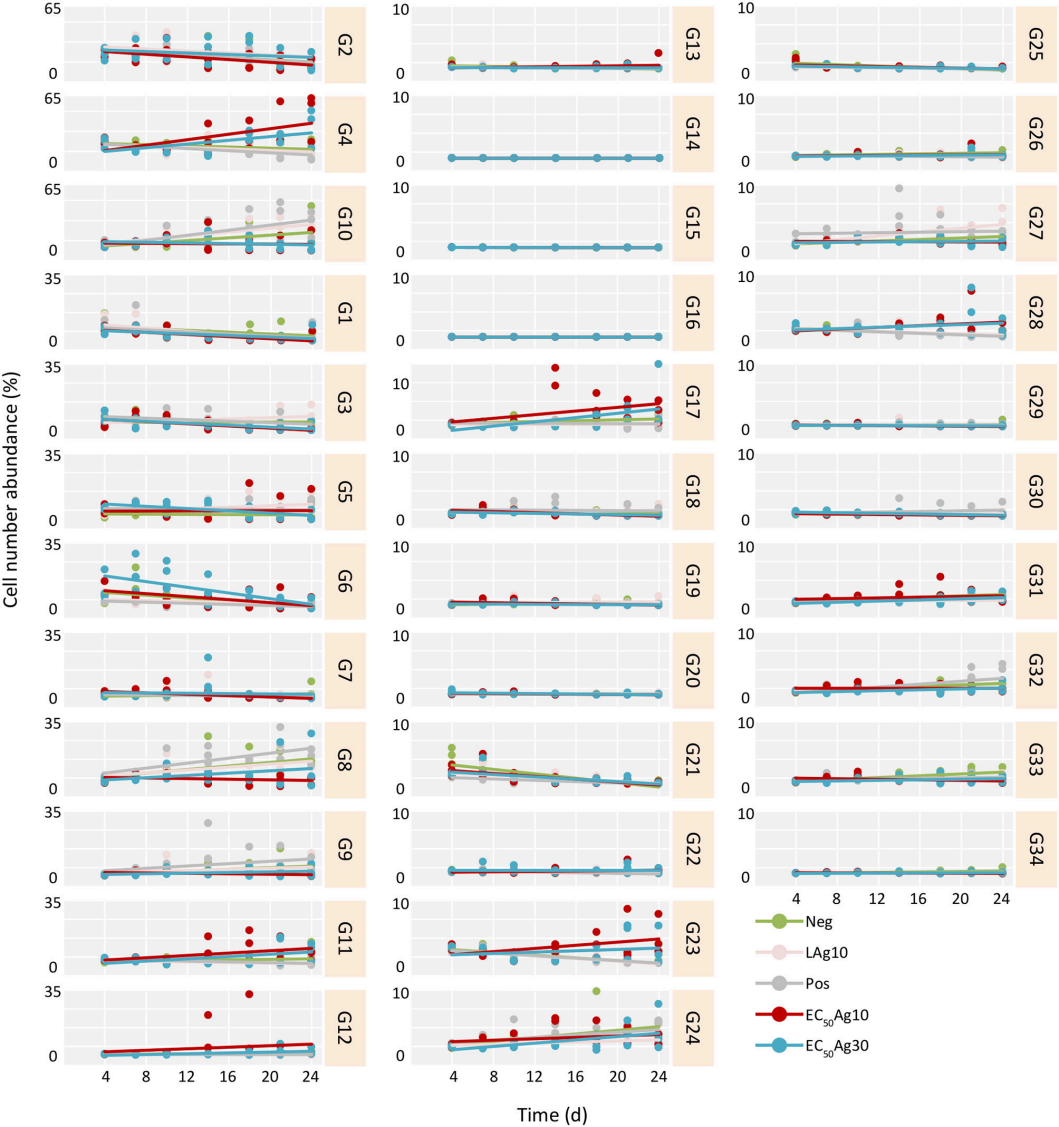
Trends in cell number increase or decrease per treatment and gate from 4 to 24 d for Setup 1 (inoculum of 0 d is excluded). The trends are estimated for all treatments and their respective triplicates: silver ion negative control (Neg), 0.1 mg/L AgNP-10 (LAg10), silver ion positive control of 0.25 mg/L AgNO_3_ (Pos), 2.25 mg/L AgNP-10 (EC_50_Ag10), and 7.13 mg/L AgNP-30 (EC_50_Ag30). The slope (*k*) of each trend is given in Table S3.

Yet, if the AgNPs affected all cells in a community equally or acted selectively on particular subcommunities still remained to be clarified. Therefore, trends of cell abundance variations for each gate per treatment were estimated from 4 d (after the adaptation) to 24 d, to mark the influenced gates (Figure 2 and Table S3). Typically, for the controls and the low AgNP concentration, similar subcommunities (G8–G10) dominated the whole communities. In contrast, at EC_50_ values of AgNPs (10 and 30 nm) the whole communities were dominated by other subcommunities, e.g., G4 and G11. At the same EC_50_ values of AgNPs (10 and 30 nm) decreases in cell abundances were strong in some of the initially highly abundant subcommunities such as G1–G3, and G6. The slopes (*k*) of trends per treatment and gate, calculated for the time range from 4 to 24 d, highlighted this development (Figure 2 and Table S3). For example, the EC_50_Ag30 *k-*values were for G1: −0.21, G2: −0.37, G3: −0.25, and G6: −0.75. Contrarily, cell abundances increased for G4 from 3.1 ± 0.1 to 46.5 ± 23.8% and for G11 from 0.8 ± 0.1 to 8.3 ± 4.8% (with *k-*values of 0.92 and 0.31, respectively) within 20 d under EC_50_Ag30 treatment. These data indicate that AgNPs at EC_50_ values selectively affect specific subcommunities.

Further, the effect of each treatment on the cells was calculated by using Spearman's correlation coefficients (rho). Only correlations with rho ≥ | 0.4 | were regarded as strong (Table S4). For the controls and the low AgNP concentration treatment, the dominant gates G8–G10 showed always strong positive rho values. Instead, under EC_50_AgNP treatments, other subcommunities emerged (G4 and G11) with solid positive rho values. Contrarily, under the same conditions, G3 was one of those gates that lost cell numbers during EC_50_AgNP treatments which is verified by highly significant and strongly negative rho values.

### Diversity Values of Microbial Communities Influenced by Silver

Since AgNPs at EC_50_ values led to more distinct community structure changes, their influences on the community diversity were further investigated via two ecological metrics based on the cytometric gate information: cytometric α-diversity and intra-community β-diversity.

**Figure 3:**
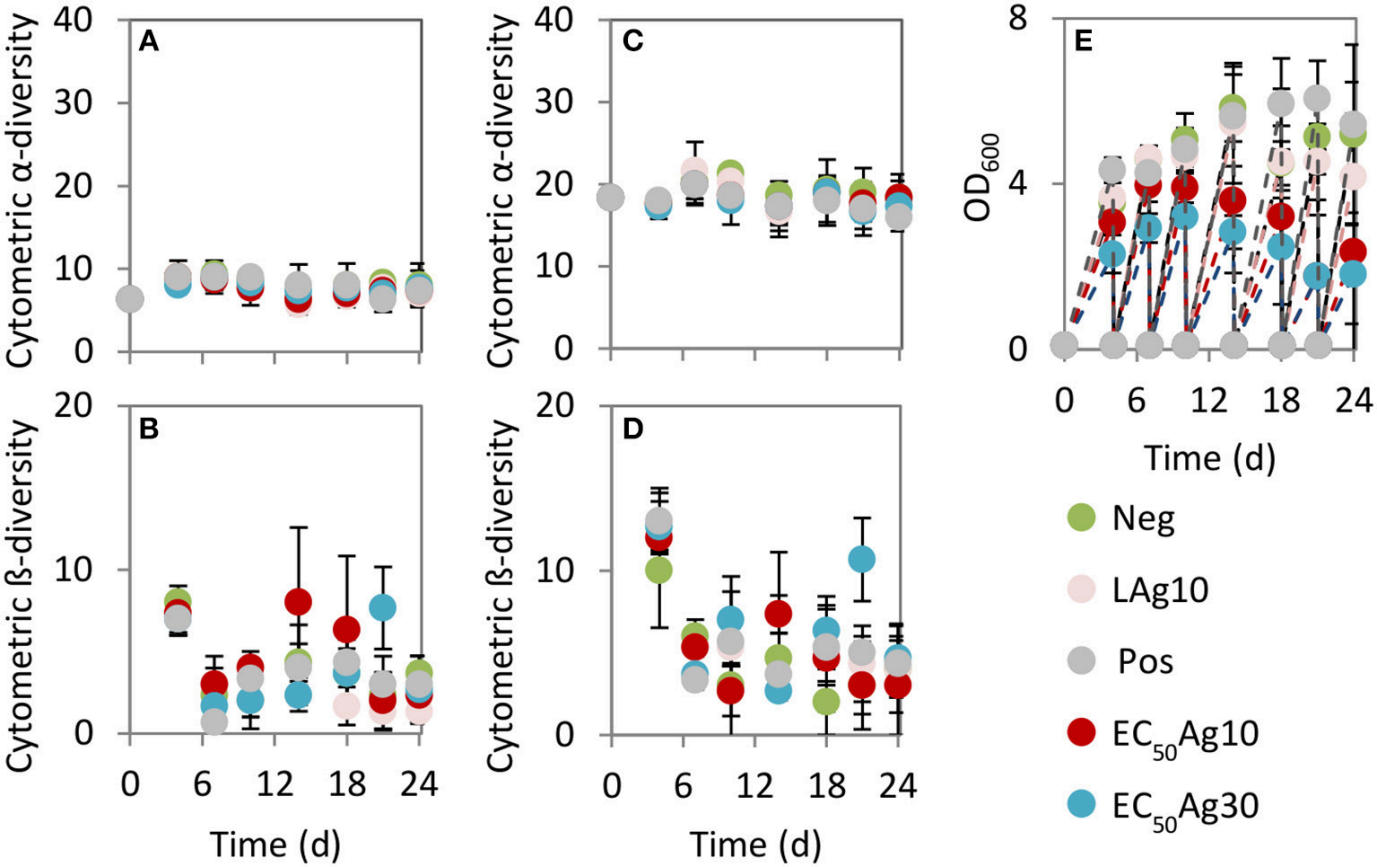
Cytometric diversity metrics **(A–D)** and biomass production **(E)**. Data are shown for Setup 1 for silver ion negative control (Neg), 0.1 mg/L AgNP-10 (LAg10), 2.25 mg/L AgNP-10 (EC_50_Ag10), 7.13 mg/L AgNP-30 (EC_50_Ag30), silver ion positive control of 0.25 mg/L AgNO_3_ (Pos). **(A)** Cytometric α-diversity values (threshold of 2.9%), **(B)** Cytometric intra-community β-diversity values (threshold of 2.9%), **(C)** Cytometric α-diversity values (at lower threshold of 0.71%), **(D)** Cytometric intra-community β-diversity values (at lower threshold of 0.71%). Error bars are sample standard deviations from three parallel experiments. Comparable data for Setup 2 are shown in Figure S4.

The cytometric α-diversity values were constant between 6 and 9 for the silver-free and silver-confined samples over 24 d (Figure 3A). However, the cytometric intra-community β-diversity showed obvious fluctuations. Highest values were found after 4 d (values between 7 and 8) for all silver-free and silver-confined samples, which implied an adaptation process from the wastewater inoculum to the sequenced-batch cultivation conditions (Figure 3B). Afterwards, the negative and the positive controls and low AgNP concentration treatment showed a stable intra-community β-diversity (values between 1 and 4). Instead, AgNPs at EC_50_ values caused higher intra-community β-diversity variations (values between 2 and 8), which suggested serious community differences between sampling days.

By choosing a lower threshold of 0.71% to determine Hill number D_q = 0_, which was adjusted according to the threshold set by the 16S rRNA gene amplicon sequencing data, the cytometric α-diversity and intra-community β-diversity values showed the same trends (Figures 3C,D). Although more subcommunities were included in the richness analysis (values between 16 and 21) (Figure 3C) in comparison to the higher average cell abundance threshold per gate of 2.9%, the general trend in community behavior did not change.

In addition, the production of biomass, measured by OD_600_, was significantly decreased at EC_50_AgNP treatments by a factor of 2.2 (EC_50_Ag10) and 2.9 (EC_50_Ag30, Figure 3E) when being compared to the negative control. In contrast, low AgNP concentration treatment (factor 1.2) and the positive control (factor 1.0) did not cause huge reduction in biomass. Comparable results for Setup 2 are presented in Figure S4.

### 16S rRNA Gene Amplicon Sequencing to Confirm the Flow Cytometric Data

To confirm the cytometric results of whole microbial community structure changes caused by AgNPs at EC_50_ values, selected samples from Setup 1 were further identified by using 16S rRNA gene amplicon sequencing. Therefore, samples were chosen from 7 d, where the adaptation of the microbial community to the new condition was solid, and from 24 d, where the treatment with AgNPs was the longest. To identify the species of subcommunities that strongly responded positively or negatively to AgNPs at EC_50_ concentrations (Figure 2, Table S4), subcommunities G4, G11 and G3 from 24 and 7 d were sorted and sequenced. For each sequenced sample, one of the triplicates from either the silver-free and silver-confined batches was chosen as is described in Table S2.

**Figure 4:**
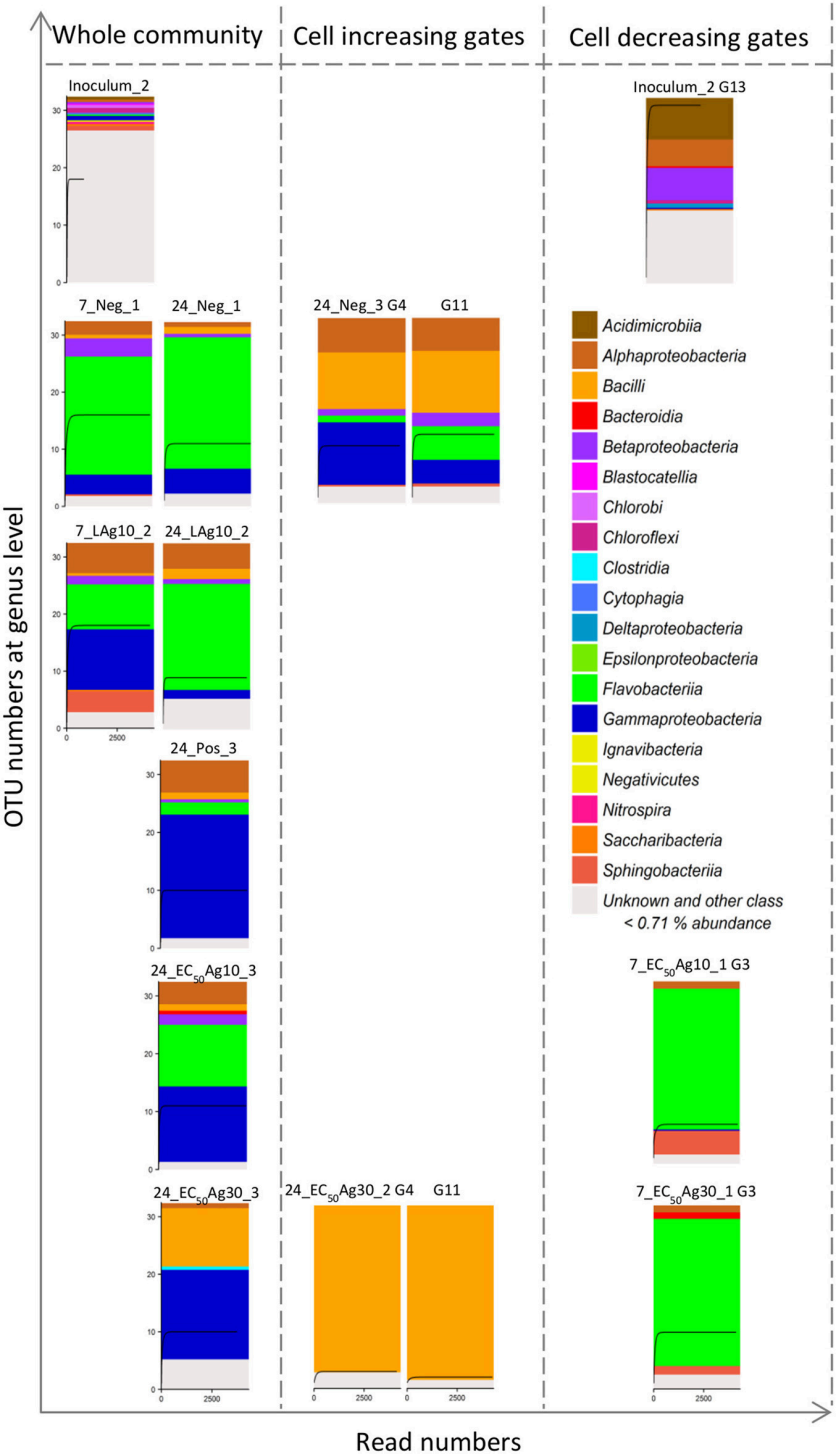
16S rRNA gene amplicon sequencing of 15 samples including 8 whole community samples and 7 sorted subcommunities. Data are shown for Setup 1. The 8 whole community samples included inoculum, silver ion negative control (Neg, 7 and 24 d) and 0.1 mg/L AgNP-10 (LAg10, 7 and 24 d), silver ion positive control (Pos, 24 d), 2.25 mg/L AgNP-10 (EC_50_Ag10, 24 d), 7.13 mg/L AgNP-30 (EC_50_Ag30, 24 d). The 7 sorted subcommunities included the dominant G13 in the inoculum, the positive correlating G4 and G11, and negative correlating G3 with the duration of silver exposure. Each class above a threshold of 0.71% is presented by a color. The rarefaction curve inside each histogram represents the number of genera. Details of class and relative genera list with abundances are shown in Table S5.

The bacterial compositions of sequenced samples are presented in Figure 4. The community's diversity was resolved at the class level. Their diversity decreased due to the adaptation from the wastewater inoculum (12 OTUs above threshold of 0.71%, sample Inoculum_2) to batch cultivations (4–7 OTUs above threshold of 0.71%, samples on 7 and 24 d, Figure 4), which, however, remained constant as was shown by the cytometric α-diversity (Figure 3C).

Similar to the cytometric data, the sequencing data revealed changes in microbial community structures. The wastewater inoculum represented unique phylotypes (e.g., *Acidimicrobiia, Blastocatellia, Cytophagia, Chlorobi, Ignavibacteria, Nitrospira, Saccharibacteria*), which were absent during the sequenced-batch cultivation mode. G13, which was only highly abundant (9.8 ± 0.9%) in the inoculum, was sorted and sequenced and found to be dominated mostly by *Acidimicrobiia* (22.3%). Instead, the negative control and the sample treated by low AgNP concentration showed microbial community compositions that were different from the inoculum but similar among themselves on 24 d. Both samples were dominated by *Flavobacteriia* of 71.1 and 57.0%, respectively. The positive control was occupied by *Gammaproteobacteria* of 65.9%. The decrease of *Flavobacteriia* and increase of *Gammaproteobacteria* was also found by the EC_50_AgNP treatments: the *Flavobacteriia* decreased to 32.9% (EC_50_Ag10) and 0.0% (EC_50_Ag30), and the *Gammaproteobacteria* rose up to 40.3 and 48%, respectively (3.0- and 3.5-fold higher than the negative control on 24 d). Additionally, *Bacilli* were found with cell abundance rising up to 31.3% at EC_50_Ag30 treatment (8.4-fold higher than the negative control on 24 d). In this treatment, *Bacilli* were the main phylotypes in the sorted gates G4 and G11 with up to 91.4 and 95.4%, respectively. In the negative control these gates contained the *Bacilli* phylotype to only 40.0%. Instead, the sorted G3 contained *Flavobacteriia* with up to 80.1%.

## Discussion

The aim of this work was to study the impact of AgNPs on microbial community structure and dynamics of wastewater systems by using dense-sampling high-throughput flow cytometry and selected 16S rRNA gene amplicon sequencing. The cytometric results suggested that the investigated wastewater microbial communities tolerated low AgNP concentrations and the silver ion concentration of the positive control (AgNO_3_). The relative diversity was not affected, and the biomass production was reduced by not more than 19.9% (low AgNP concentration treatment). Contrarily, AgNPs at EC_50_ values led to clear changes in cytometric community structure, which were obvious since certain subcommunities became dominant, and a biomass loss up to 65.1% (EC_50_Ag30) was found in comparison to the negative control. This higher toxicity of AgNPs at EC_50_ values may originate from their assembly to aggregates which present potential long-term sources of silver ions and thus an enduring toxic effects in comparison to the steady level of silver ions in the positive control (AgNO_3_) (Guo et al., 2017b). The two sizes of AgNPs at EC_50_ values showed no effects on cytometric α-diversity values but comparable biomass reduction on wastewater microbial communities. This could be due to the similar amount of dissolved silver ions from the two sizes of nanoparticles at EC_50_ values as was discussed earlier (Guo et al., 2017b). Instead, the cytometric intra-community β-diversity values varied between EC_50_Ag10 and EC_50_Ag30 concentrations. As the complex wastewater community succession was not synchronous between the different treatments, it can be suggested that the species replacement was divergent and therefore their responses different.

Despite the profound loss in biomass caused by AgNPs at EC_50_ concentrations, some phylotypes persisted. On the one hand, this persistence may contribute to an intact functionality of the wastewater microbial community since functions are usually redundant (Liang et al., 2010). On the other hand in this study phylotypes that were found to be persistent may present potential problems to the ecosystems. *Bacilli* were found to dominate the whole community with up to 31.3%. The subcommunity G4 seems to be a marker gate for this class. *Bacilli* and *Gammaproteobacteria* which also increased proportionally in comparison to the negative control are known to potentially serve as reservoirs of antibiotic resistance genes (Xiong et al., 2015). It has been reported that heavy metals, such as silver, can promote the selection and enrichment of antibiotic resistance genes (Gullberg et al., 2014; Chen et al., 2015; Li et al., 2017), and silver resistant genes that encode and induce efflux mechanisms have been found in members of the class *Bacilli* and *Gammaproteobacteria* (Babu et al., 2011; Sütterlin et al., 2014). Such mechanisms may support the persistence of *Bacilli* and *Gammaproteobacteria* and contributed to their dominance over other species by EC_50_AgNP treatments. Furthermore, many members of *Bacilli* are known to be able to form spores when the environment is not supporting growth. These spores are extremely resistant to most environmental stress factors. They have little or no metabolic activity and thus are considered as dormant. The dormancy state could last until the environmental conditions can be tolerated by the microorganisms and growth is possible again (Setlow, 2014). It is suggested that tolerant/resistant species are bound to increase in cell numbers by outcompeting sensitive ones in natural communities (Xavier, 2014), an event that was also found for the *Bacilli* and *Gammaproteobacteria* in our sequenced-batch grown wastewater communities. Many well-known pathogens are included in both classes of bacteria, e.g., *Bacillus anthracis*, the cause of anthrax (Spencer, 2003), and *Acinetobacter* (Table S5) as an opportunistic pathogen affecting people with compromised immune systems (Antunes et al., 2014).

However, the loss of phylotypes was also detected, like *Flavobacteriia*, which is the predominant class in *Bacteroidetes* phylum from the sequencing data set. This class dominated in the negative control but decreased in cell abundance in the other setups, especially in EC_50_Ag30 treatment. The subcommunity G3, although showing generally low cell abundances, seems to be a marker gate for this class. *Flavobacteriia* are usually widely distributed in soil and water. It has been reported that *Flavobacterium* sp. supported granule formation in activated sludge due to the production of extracellular polymeric substances (Li et al., 2014). The proportional decrease of *Flavobacteriia* might therefore lead to a loss in the stability of the sludge. This finding is consistent with other published results which showed that *Bacteroidetes* are especially affected by nanoparticles. They are present mainly on the surface of granular sludge or sediment and can, therefore, be easily targeted by AgNPs (Sun et al., 2013; Yang et al., 2016).

Notably, the proportional loss or increase in cell abundance in specific gates and thus their contribution to the whole community fingerprint are sensitive indicators for non-toxic or toxic environments to microbial communities. The use of ecological metrics allowed a fast, continuous and cost-effective screening of the microbial community dynamics which enabled to uncover subcommunities that may serve as indicators for toxic situations. By cell sorting and sequencing of respective gates, the phylogenetic affiliation of the contained members was resolved and, in a future step, subcommunity metagenomics may help to reveal e.g., possibly involved resistance genes. Therefore, this high-throughput technique improves environmental risk assessment and may assess toxic concentration levels e.g., of silver on natural or managed microbial communities, as was done in this study, in a more profound way than classical measurement, e.g., EC_50_ determination will allow.

## Author Contributions

YG designed and conducted the experiments, evaluated the data and wrote the paper. NC analyzed community and sorted samples by using 16S rRNA gene amplicon Illumina MiSeq sequencing, evaluated those data, and contributed to writing. FS analyzed samples by using flow cytometry, helped to evaluate the data, and contributed to experimental design and writing. RG contributed to 16S rRNA gene amplicon Illumina MiSeq sequencing. HH contributed to writing. SM designed the experiments, evaluated the data, and wrote the paper.

### Conflict of Interest Statement

The authors declare that the research was conducted in the absence of any commercial or financial relationships that could be construed as a potential conflict of interest.

## Footnotes

^1^http://ec.europa.eu/eurostat/statistics-explained/index.php?title = File:Sewage_sludge_disposal_from_urban wastewater_treatment,_by_type_of_treatment,_2015_(%25_of_total_mass)_V2.png

^2^http://www.gesetze-im-internet.de/abwv/AbwV.pdf

^3^https://flowrepository.org/id/FR-FCM-ZYD7-8

^4^https://bioconductor.org/packages/release/bioc/html/flowCyBar.html

## References

[B1] Antunes L. C. Visca P. Towner K. J. (2014). Acinetobacter baumannii: evolution of a global pathogen. Pathog. Dis. 71, 292–301. 10.1111/2049-632X.12125 24376225

[B2] Babu M. M. Sridhar J. Gunasekaran P. (2011). Global transcriptome analysis of *Bacillus cereus* ATCC 14579 in response to silver nitrate stress. J. Nanobiotechnol. 9:49. 10.1186/1477-3155-9-49 22071005 PMC3247866

[B3] Barton L. E. Auffan M. Durenkamp M. McGrath S. Bottero J.-Y. Wiesner M. R. (2015). Monte Carlo simulations of the transformation and removal of Ag, TiO2, and ZnO nanoparticles in wastewater treatment and land application of biosolids. Sci. Total Environ. 511, 535–543. 10.1016/j.scitotenv.2014.12.056 25585156

[B4] Benn T. M. Westerhoff P. (2008). Nanoparticle silver released into water from commercially available sock fabrics. Environ. Sci. Technol. 42, 4133–4139. 10.1021/es7032718 18589977

[B5] Bokulich N. A. Subramanian S. Faith J. J. Gevers D. Gordon J. I. Knight R.. (2012). Quality-filtering vastly improves diversity estimates from Illumina amplicon sequencing. Nat. Methods 10, 57–59. 10.1038/nmeth.2276 23202435 PMC3531572

[B6] Bondarenko O. Ivask A. Käkinen A. Kurvet I. Kahru A. (2013). Particle-cell contact enhances antibacterial activity of silver nanoparticles. PLoS ONE 8:e64060. 10.1371/journal.pone.0064060 23737965 PMC3667828

[B7] Chen S. Li X. Sun G. Zhang Y. Su J. Ye J. (2015). Heavy metal induced antibiotic resistance in bacterium LSJC7. Int. J. Mol. Sci. 16, 23390–23404. 10.3390/ijms161023390 26426011 PMC4632705

[B8] Chen X. Schluesener H. J. (2008). Nanosilver: a nanoproduct in medical application. Toxicol. Lett. 176, 1–12. 10.1016/j.toxlet.2007.10.004 18022772

[B9] Choubert J. M. Martin Ruel S. Esperanza M. Budzinski H. Miège C. Lagarrigue C.. (2011). Limiting the emissions of micro-pollutants: what efficiency can we expect from wastewater treatment plants? Water Sci. Technol. 63, 57–65. 10.2166/wst.2011.009 21245554

[B10] Domingo G. Bracale M. Vannini C. (2019). Phytotoxicity of silver nanoparticles to aquatic plants, algae, and microorganisms. Nanomater. Plants Algae Microorgan. 2, 143–168. 10.1016/B978-0-12-811488-9.00008-1

[B11] Edgar R. C. Haas B. J. Clemente J. C. Quince C. Knight R. (2011). UCHIME improves sensitivity and speed of chimera detection. Bioinformatics 27, 2194–2200. 10.1093/bioinformatics/btr381 21700674 PMC3150044

[B12] Gottschalk F. Sonderer T. Scholz R. W. Nowack B. (2009). Modeled environmental concentrations of engineered nanomaterials (TiO2, ZnO, Ag, CNT, Fullerenes) for different regions. Environ. Sci. Technol. 43, 9216–9222. 10.1021/es9015553 20000512

[B13] Gullberg E. Albrecht L. M. Karlsson C. Sandegren L. Andersson D. I. (2014). Selection of a multidrug resistance plasmid by sublethal levels of antibiotics and heavy metals. mBio 5, e01918–14. 10.1128/mBio.01918-14 25293762 PMC4196238

[B14] Günther S. Faust K. Schumann J. Harms H. Raes J. Müller S. (2016). Species-sorting and mass-transfer paradigms control managed natural metacommunities. Environ. Microbiol. 18, 4862–4877. 10.1111/1462-2920.13402 27338005

[B15] Günther S. Koch C. Hübschmann T. Röske I. Müller R. A. Bley T.. (2012). Correlation of community dynamics and process parameters as a tool for the prediction of the stability of wastewater treatment. Environ. Sci. Technol. 46, 84–92. 10.1021/es2010682 21805973

[B16] Guo Y. Baumgart S. Stärk H.-J. Harms H. Müller S. (2017a). Mass cytometry for detection of silver at the bacterial single cell level. Front. Microbiol. 8:1326. 10.3389/fmicb.2017.01326 28769897 PMC5511850

[B17] Guo Y. Stärk H. J. Hause G. Schmidt M. Harms H. Wick L. Y. (2017b). Heterogenic response of prokaryotes toward silver nanoparticles and ions is facilitated by phenotypes and attachment of silver aggregates to cell surfaces: heterogenic response of prokaryotes toward AgNPs. Cytometry A 91, 775–784. 10.1002/cyto.a.23055 28110496

[B18] Hendren C. O. Badireddy A. R. Casman E. Wiesner M. R. (2013). Modeling nanomaterial fate in wastewater treatment: Monte Carlo simulation of silver nanoparticles (nano-Ag). Sci. Total Environ. 449, 418–425. 10.1016/j.scitotenv.2013.01.078 23454703

[B19] Herlemann D. P. Labrenz M. Jürgens K. Bertilsson S. Waniek J. J. Andersson A. F. (2011). Transitions in bacterial communities along the 2000 km salinity gradient of the Baltic Sea. ISME J. 5, 1571–1579. 10.1038/ismej.2011.41 21472016 PMC3176514

[B20] Hill M. O. (1973). Diversity and evenness: a unifying notation and its consequences. Ecology 54, 427–432. 10.2307/1934352

[B21] Jelic A. Gros M. Ginebreda A. Cespedes-Sánchez R. Ventura F. Petrovic M.. (2011). Occurrence, partition and removal of pharmaceuticals in sewage water and sludge during wastewater treatment. Water Res. 45, 1165–1176. 10.1016/j.watres.2010.11.010 21167546

[B22] Jin X. Li M. Wang J. Marambio-Jones C. Peng F. Huang X.. (2010). High-throughput screening of silver sanoparticle stability and bacterial inactivation in aquatic media: influence of specific ions. Environ. Sci. Technol. 44, 7321–7328. 10.1021/es100854g 20873875

[B23] Kim J. S. Kuk E. Yu K. N. Kim J.-H. Park S. J. Lee H. J.. (2007). Antimicrobial effects of silver nanoparticles. Nanomed-Nanotechnol. 3, 95–101. 10.1016/j.nano.2006.12.001 17379174

[B24] Koch C. Günther S. Desta A. F. Hübschmann T. Müller S. (2013). Cytometric fingerprinting for analyzing microbial intracommunity structure variation and identifying subcommunity function. Nat. Protoc. 8, 190–202. 10.1038/nprot.2012.149 23288319

[B25] Koch C. Harnisch F. Schröder U. Müller S. (2014). Cytometric fingerprints: evaluation of new tools for analyzing microbial community dynamics. Front. Microbiol. 5:273. 10.3389/fmicb.2014.00273 24926290 PMC4044693

[B26] Li J. Ding L.-B. Cai A. Huang G.-X. Horn H. (2014). Aerobic sludge granulation in a full-scale sequencing batch reactor. BioMed Res. Int. 2014, 1–12. 10.1155/2014/268789 24822190 PMC4009315

[B27] Li L. Stoiber M. Wimmer A. Xu Z. Lindenblatt C. Helmreich B.. (2016). To what extent can full-scale wastewater treatment plant effluent influence the occurrence of silver-based nanoparticles in surface waters? Environ. Sci. Technol. 50, 6327–6333. 10.1021/acs.est.6b00694 27228366

[B28] Li L.-G. Xia Y. Zhang T. (2017). Co-occurrence of antibiotic and metal resistance genes revealed in complete genome collection. ISME J. 11, 651–662. 10.1038/ismej.2016.155 27959344 PMC5322307

[B29] Liang Z. Das A. Hu Z. (2010). Bacterial response to a shock load of nanosilver in an activated sludge treatment system. Water Res. 44, 5432–5438. 10.1016/j.watres.2010.06.060 20638703

[B30] Liu J. Hurt R. H. (2010). Ion release kinetics and particle persistence in aqueous nano-silver colloids. Environ. Sci. Technol. 44, 2169–2175. 10.1021/es9035557 20175529

[B31] Liu Z. Cichocki N. Hübschmann T. Süring C. Ofiţeru I. D. Sloan W. T.. (2018). Neutral mechanisms and niche differentiation in steady-state insular microbial communities revealed by single cell analysis. Environ. Microbiol. 10.1111/1462-2920.14437. [Epub ahead of print]. 30289191 PMC7379589

[B32] Lok C.-N. Ho C.-M. Chen R. He Q.-Y. Yu W.-Y. Sun H.. (2007). Silver nanoparticles: partial oxidation and antibacterial activities. JBIC J. Biol. Inorg. Chem. 12, 527–534. 10.1007/s00775-007-0208-z 17353996

[B33] Marambio-Jones C. Hoek E. M. V. (2010). A review of the antibacterial effects of silver nanomaterials and potential implications for human health and the environment. J. Nanoparticle Res. 12, 1531–1551. 10.1007/s11051-010-9900-y

[B34] Oh S. Y. Sung H. K. Park C. Kim Y. (2015). Biosorptive removal of bare-, citrate-, and PVP-coated silver nanoparticles from aqueous solution by activated sludge. J. Ind. Eng. Chem. 25, 51–55. 10.1016/j.jiec.2014.10.012

[B35] Pesce S. Ghiglione J.-F. Martin-Laurent F. (2017). Microbial communities as ecological indicators of ecosystem recovery following chemical pollution. Microbial Ecotoxicol. 227–250. 10.1007/978-3-319-61795-4_10

[B36] Props R. Monsieurs P. Mysara M. Clement L. Boon N. (2016). Measuring the biodiversity of microbial communities by flow cytometry. Methods Ecol. Evol. 7, 1376–1385. 10.1111/2041-210X.12607

[B37] Pulit-Prociak J. Banach M. (2016). Silver nanoparticles – a material of the future…? Open Chem. 14, 76–91. 10.1515/chem-2016-0005

[B38] Quast C. Pruesse E. Yilmaz P. Gerken J. Schweer T. Yarza P.. (2013). The SILVA ribosomal RNA gene database project: improved data processing and web-based tools. Nucleic Acids Res. 41, D590–D596. 10.1093/nar/gks1219 23193283 PMC3531112

[B39] Rai M. Yadav A. Gade A. (2009). Silver nanoparticles as a new generation of antimicrobials. Biotechnol. Adv. 27, 76–83. 10.1016/j.biotechadv.2008.09.002 18854209

[B40] Reidy B. Haase A. Luch A. Dawson K. Lynch I. (2013). Mechanisms of silver nanoparticle release, transformation and toxicity: a critical review of current knowledge and recommendations for future studies and applications. Materials 6, 2295–2350. 10.3390/ma6062295 28809275 PMC5458943

[B41] Schloss P. D. Westcott S. L. Ryabin T. Hall J. R. Hartmann M. Hollister E. B.. (2009). Introducing mothur: open-source, platform-independent, community-supported software for describing and comparing microbial communities. Appl. Environ. Microbiol. 75, 7537–7541. 10.1128/AEM.01541-09 19801464 PMC2786419

[B42] Setlow P. (2014). Germination of spores of Bacillus species: what we know and do not know. J. Bacteriol. 196, 1297–1305. 10.1128/JB.01455-13 24488313 PMC3993344

[B43] Singer E. Andreopoulos B. Bowers R. M. Lee J. Deshpande S. Chiniquy J.. (2016). Next generation sequencing data of a defined microbial mock community. Sci. Datas 3:160081. 10.1038/sdata.2016.81 27673566 PMC5037974

[B44] Spencer R. C. (2003). Bacillus anthracis. J. Clin. Pathol. 56, 182–187. 10.1136/jcp.56.3.182 12610093 PMC1769905

[B45] Storck V. Nikolaki S. Perruchon C. Chabanis C. Sacchi A. Pertile G.. (2018). Lab to field assessment of the ecotoxicological impact of chlorpyrifos, isoproturon, or tebuconazole on the diversity and composition of the soil bacterial community. Front. Microbiol. 9:1412. 10.3389/fmicb.2018.01412 30008705 PMC6034002

[B46] Sun X. Sheng Z. Liu Y. (2013). Effects of silver nanoparticles on microbial community structure in activated sludge. Sci. Total Environ. 443, 828–835. 10.1016/j.scitotenv.2012.11.019 23246663

[B47] Suresh A. K. Pelletier D. A. Wang W. Moon J.-W. Gu B. Mortensen N. P.. (2010). Silver nanocrystallites: biofabrication using *Shewanella oneidensis*, and an evaluation of their comparative toxicity on Gram-negative and Gram-positive bacteria. Environ. Sci. Technol. 44, 5210–5215. 10.1021/es903684r 20509652

[B48] Sütterlin S. Edquist P. Sandegren L. Adler M. Tängdén T. Drobni M.. (2014). Silver resistance genes are overrepresented among *Escherichia coli* isolates with CTX-M production. Appl. Environ. Microbiol. 80, 6863–6869. 10.1128/AEM.01803-14 25128339 PMC4249003

[B49] Takahashi S. Tomita J. Nishioka K. Hisada T. Nishijima M. (2014). Development of a prokaryotic universal primer for simultaneous analysis of bacteria and archaea using next-generation sequencing. PLoS ONE 9:e105592. 10.1371/journal.pone.0105592 25144201 PMC4140814

[B50] Tiede K. Boxall A. B. A. Wang X. Gore D. Tiede D. Baxter M. (2010). Application of hydrodynamic chromatography-ICP-MS to investigate the fate of silver nanoparticles in activated sludge. J. Anal. Atmos. Spectr. 25, 1149–1154. 10.1039/b926029c

[B51] Vance M. E. Kuiken T. Vejerano E. P. McGinnis S. P. Hochella M. F. Rejeski D.. (2015). Nanotechnology in the real world: redeveloping the nanomaterial consumer products inventory. Beilstein J. Nanotechnol. 6, 1769–1780. 10.3762/bjnano.6.181 26425429 PMC4578396

[B52] Visnapuu M. Joost U. Juganson K. Künnis-Beres K. Kahru A. Kisand V.. (2013). Dissolution of silver nanowires and nanospheres dictates their toxicity to *Escherichia coli*. BioMed. Res. Int. 2013:819252. 10.1155/2013/819252 24024212 PMC3762159

[B53] Voelker D. Schlich K. Hohndorf L. Koch W. Kuehnen U. Polleichtner C.. (2015). Approach on environmental risk assessment of nanosilver released from textiles. Environ. Res. 140, 661–672. 10.1016/j.envres.2015.05.011 26073205

[B54] von Moos N. Slaveykova V. I. (2014). Oxidative stress induced by inorganic nanoparticles in bacteria and aquatic microalgae – state of the art and knowledge gaps. Nanotoxicology 8, 605–630. 10.3109/17435390.2013.809810 23738945

[B55] Wijnhoven S. W. P. Peijnenburg W. J. G. M. Herberts C. A. Hagens W. I. Oomen A. G. Heugens E. H. W. (2009). Nano-silver – a review of available data and knowledge gaps in human and environmental risk assessment. Nanotoxicology 3, 109–138. 10.1080/17435390902725914

[B56] Xavier J. B. (2014). Social interaction in synthetic and natural microbial communities. Mol. Syst. Biol. 7, 483–483. 10.1038/msb.2011.16 21487402 PMC3101950

[B57] Xiong W. Sun Y. Ding X. Wang M. Zeng Z. (2015). Selective pressure of antibiotics on ARGs and bacterial communities in manure-polluted freshwater-sediment microcosms. Front. Microbiol. 6:194. 10.3389/fmicb.2015.00194 25814986 PMC4356103

[B58] Xiu Z. M. Ma J. Alvarez P. J. J. (2011). Differential effect of common ligands and molecular oxygen on antimicrobial activity of silver nanoparticles versus silver ions. Environ. Sci. Technol. 45, 9003–9008. 10.1021/es201918f 21950450

[B59] Xiu Z. M. Zhang Q. Puppala H. L. Colvin V. L. Alvarez P. J. J. (2012). Negligible particle-specific antibacterial activity of silver nanoparticles. Nano Lett. 12, 4271–4275. 10.1021/nl301934w 22765771

[B60] Yang J.-L. Li Y.-F. Liang X. Guo X.-P. Ding D.-W. Zhang D.. (2016). Silver nanoparticles impact biofilm communities and mussel settlement. Sci. Rep. 6:37406. 10.1038/srep37406 27869180 PMC5116650

[B61] Yoon K.-Y. Hoon Byeon J. Park J.-H. Hwang J. (2007). Susceptibility constants of *Escherichia coli* and *Bacillus subtilis* to silver and copper nanoparticles. Sci. Total Environ. 373, 572–575. 10.1016/j.scitotenv.2006.11.007 17173953

