# Supplementary material for: AgNPs Change Microbial Community Structures of Wastewater
Source: Front Microbiol. 2019 Jan 8;9:3211. doi: 10.3389/fmicb.2018.03211 (PMC6331452; doi:10.3389/fmicb.2018.03211)
Supplement: Supplementary file 1 [file Data_Sheet_1.PDF]

Supplementary Materials

AgNPs change microbial community structures of wastewater

Yuting Guo[1], Nicolas Cichocki[1], Florian Schattenberg[1], Robert Geffers[2], Hauke Harms[1], Susann Müller[1]*

1. Department of Environmental Microbiology, Helmholtz Centre for Environmental Research, Leipzig, Germany

2. Research Group Genome Analysis, Helmholtz Centre for Infection Research, Braunschweig, Germany

*Correspondence to: Susann Müller, E-mail: susann.mueller@ufz.de

Table S1 2

Table S2 2

Figure S1 3

Figure S2 4

Figure S3 5

Table S3 6

Table S4 7

Figure S4 8

Sequencing workflow 9

Figure S5 12

Figure S6 12

Table S5 13

References 15

Abbreviations used in the Supplementary Materials: Neg: silver ion negative control; LAg10: 0.1 mg/L AgNP-10; EC_{50}Ag10: 2.25 mg/L AgNP-10; EC_{50}Ag30: 7.13 mg/L AgNP-30; Pos: silver ion positive control

Table S1. Compositions of solutions used in the study

Solutions	Compositions
Medium	Synthetic wastewater/peptone medium = 50/50
Phosphate buffered saline (PBS)	6 mM Na_2HPO_4, 1.8 mM NaH_2PO_4, 145 mM NaCl in bi-distilled water, pH=7
2 % paraformaldehyde (PFA)	Stock solution: 8 % PFA, in PBS, pH=7<br>2 % PFA: 1 mL stock solution + 3 mL PBS
Stock A	0.11 M citric acid, 4.1 mM Tween 20 in bi-distilled water
Stock B	0.68 µM DAPI (4',6-di-amidino-2-phenyl-indole, Sigma-Aldrich, St. Louis, USA) in 417 mM Na_2HPO_4/NaH_2PO_4 buffer (289 mM Na_2HPO_4, 128 mM NaH_2PO_4 in bi-distilled water, pH=7)
Sheath fluid	10x sheath buffer (19 mM KH_2PO_4, 38 mM KCl, 166 mM Na_2HPO_4, 1.39 M NaCl in bi-distilled water) diluted with 0.1 µm filtrated bi-distilled water to a 0.2x working solution (for cell sorting: 0.5x working solution)

Table S2. Sequenced whole community samples and sorted gates of interest from Setup 1

Whole community samples		Cell number increasing gates	Cell number decreasing gates
Inoculum_2			Inoculum_2: G13
7_Neg_1	24_Neg_1	24_Neg_3: G4, G11	
7_LAg10_2	24_LAg10_2		
	24_Pos_3		
	24_EC_{50}Ag10_3		7_ EC_{50}Ag10_1: G3
	24_EC_{50}Ag30_3	24_ EC_{50}Ag30_2: G4, G11	7_ EC_{50}Ag30_1: G3

The samples were named according to the sampling time point (7 d, 24 d), treatment (Neg, LAg10, Pos, EC_{50}Ag10, EC_{50}Ag30), and one of the triplicates (1, 2, or 3).

Figure S1. Gate-template of 34 gates from 216 samples (Setup 1: 108, Setup 2: 108), where colored gates were chosen for cell sorting and later 16S rRNA gene amplicon sequencing analysis: G4, G11 as cell number increasing gates (red); G3 as cell number decreasing gate (blue). 250,000 cells were measured within a master gate which excluded instrumental noise and beads used for alignment between samples.

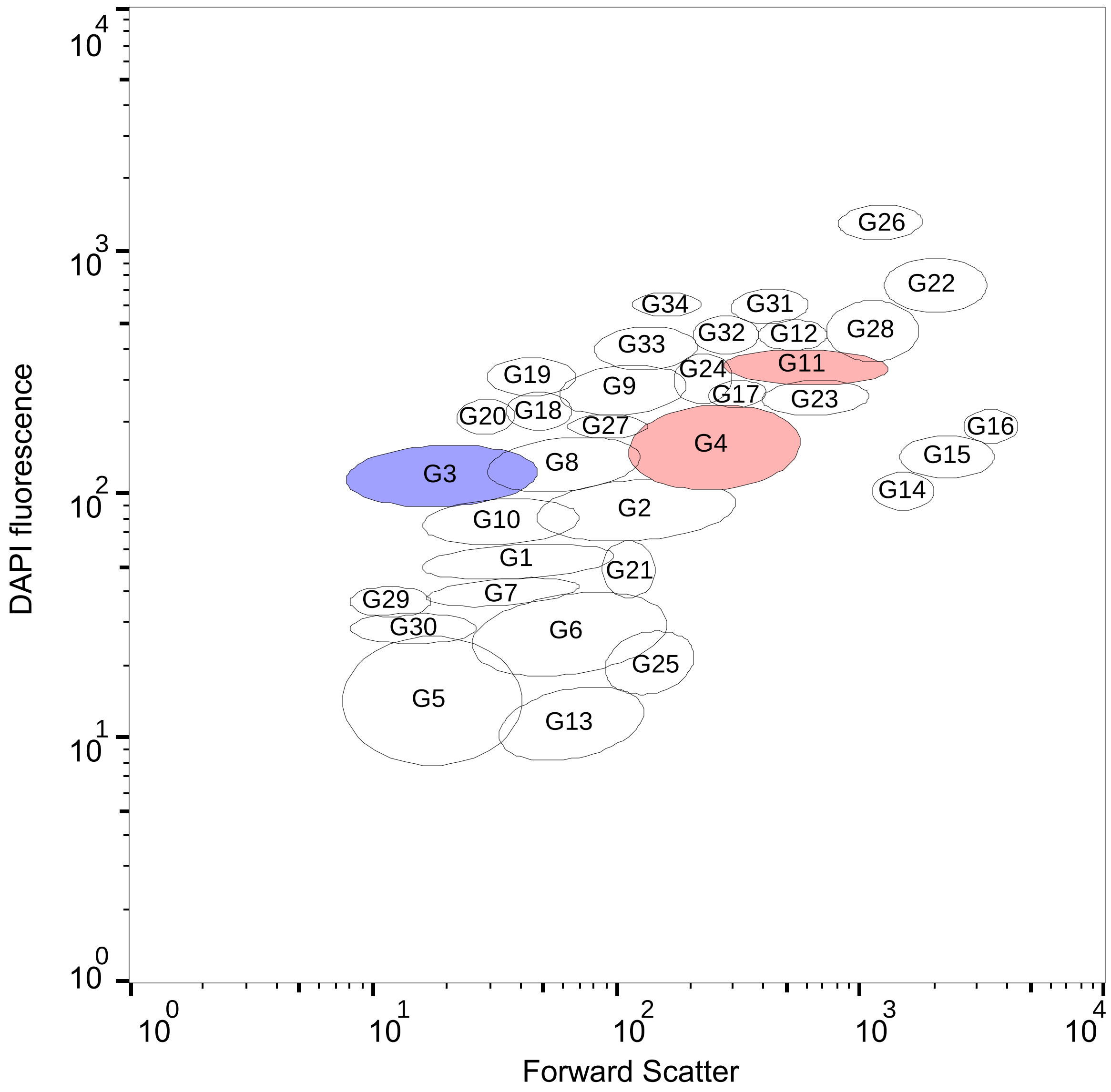

Figure S2. Changes in the cytometric community structure of Setup 1 were visualized by using the flowCyBar tool[1]. The abundance of each gate is indicated by a color gradient, where dark blue corresponds to low virtual cell abundance, and red for high virtual cell abundance (see color key). FlowCyBar was performed for each gate (34 gates) of Neg, LAg10, Pos, EC_{50}Ag10 and EC_{50}Ag30 from 0 to 24 d.

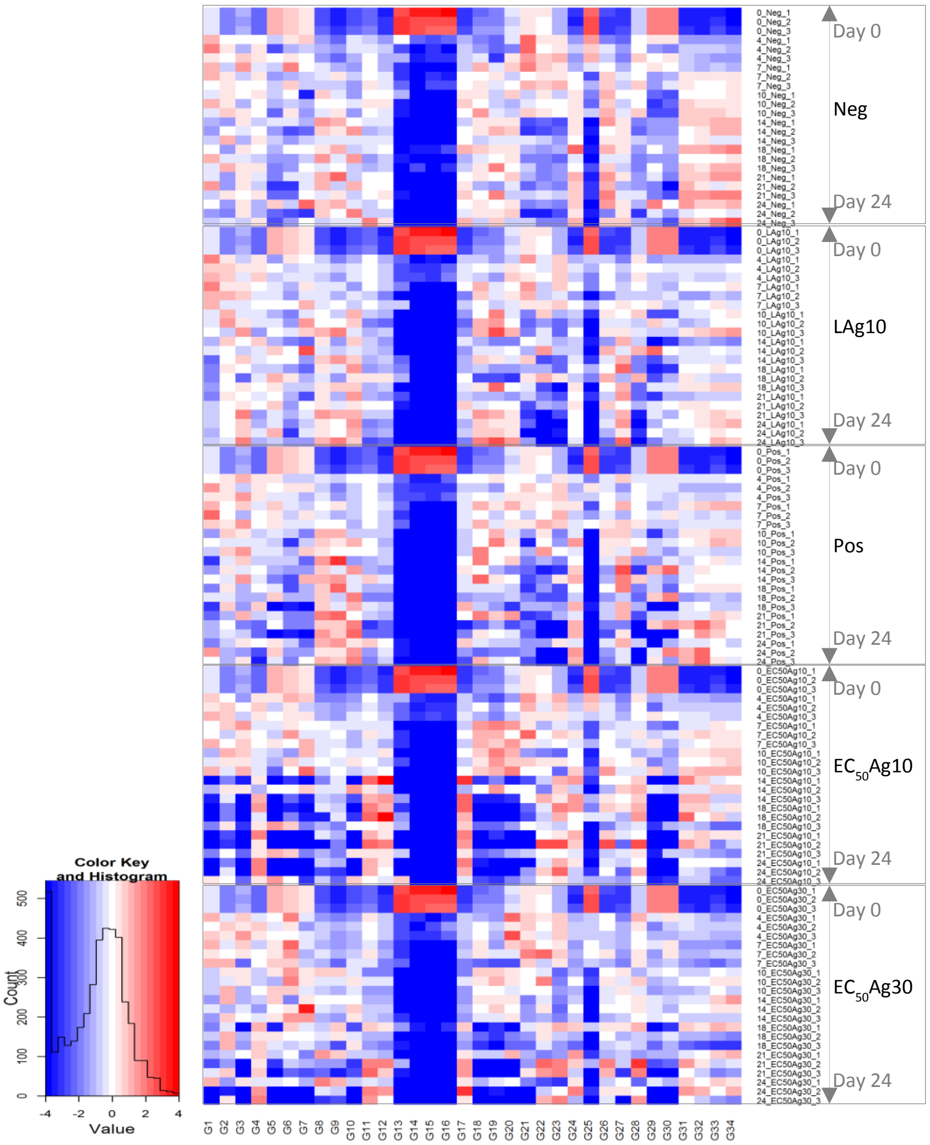

[1] https://bioconductor.org/packages/release/bioc/html/flowCyBar.html

Figure S3. Dynamic cytometric structure changes of microbial communities. Data are shown for Setup 2 (Bray-Curtis dissimilarity, stress: 0.12). Three parallel experiments were performed and shown in light, middle and dark colors: green for Neg, pink for LAg10, red for EC_{50}Ag10, blue for EC_{50}Ag30, grey for Pos. The cytometric microbial community of each sampling day is shown as a dot and the dot size increases with increasing sampling time from 0 to 24 d. All five conditions are shown together in the sub-figure at the bottom-right. Comparable data for Setup 1 are shown in Figure 1.

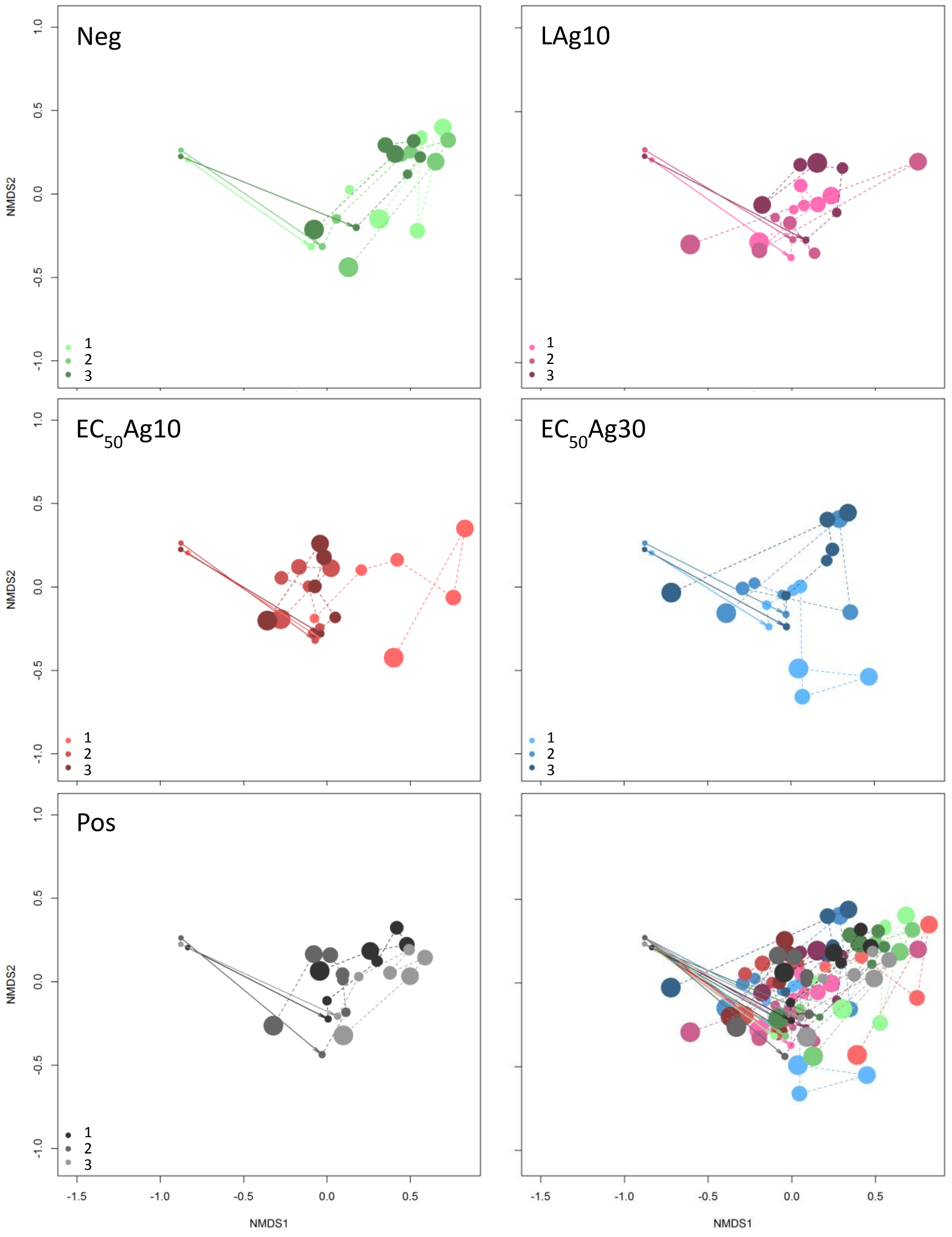

Table S3. Slopes (k) of trends per treatment and gate from 4 to 24 d for Setup 1 (inoculum of 0 d is excluded) shown in Figure 2. The slopes of trends are estimated for all treatments (Neg, LAg10, Pos, EC_{50}Ag10, EC_{50}Ag30) and their respective triplicates. For visualization, $k>0$ is shown as red dot, $k<0$ is shown as green dot.

	Slope (k)				
	Neg	LAg10	Pos	EC_{50}Ag10	EC_{50}Ag30
G1	-0.24	-0.42	-0.26	-0.29	-0.21
G2	-0.52	-0.49	-0.55	-0.67	-0.37
G3	0.00	0.17	-0.21	-0.29	-0.25
G4	-0.30	-0.37	-0.53	1.36	0.92
G5	-0.03	0.19	-0.04	0.01	-0.31
G6	-0.36	-0.13	-0.15	-0.40	-0.75
G7	0.06	-0.08	-0.06	-0.18	-0.05
G8	0.47	0.39	0.66	-0.08	0.30
G9	0.21	0.14	0.31	-0.06	0.09
G10	0.68	0.96	1.27	-0.05	-0.17
G11	0.07	-0.08	-0.10	0.31	0.31
G12	0.03	0.00	-0.01	0.21	0.11
G13	-0.03	-0.01	-0.01	0.02	0.00
G14	0.00	0.00	0.00	0.00	0.00
G15	0.00	0.00	0.00	0.00	0.00
G16	0.00	0.00	0.00	0.00	0.00
G17	0.05	0.01	-0.01	0.19	0.23
G18	0.00	0.02	-0.01	-0.05	-0.02
G19	0.02	0.01	-0.01	-0.02	-0.01
G20	0.00	0.00	-0.01	-0.01	-0.02
G21	-0.18	-0.08	-0.05	-0.12	-0.10
G22	-0.02	0.01	-0.02	0.02	0.00
G23	-0.11	-0.11	-0.10	0.12	0.05
G24	0.14	0.04	0.10	0.06	0.13
G25	-0.05	-0.01	-0.02	-0.03	-0.02
G26	0.02	0.00	-0.01	0.01	0.01
G27	0.06	0.16	0.02	-0.01	0.01
G28	-0.06	-0.05	-0.06	0.07	0.05
G29	0.01	0.00	0.00	-0.01	-0.01
G30	-0.01	0.00	0.02	-0.02	-0.02
G31	0.05	0.01	0.03	0.03	0.05
G32	0.06	0.02	0.12	0.00	0.04
G33	0.07	0.03	0.02	-0.02	0.02
G34	0.02	0.00	0.00	0.00	0.01

Table S4. Determination of Spearman's correlation coefficients (rho) to show the effect of AgNP treatment on each gate in a time range from 4 d to 24 d. These data were estimated for all treatments (Neg, LAg10, Pos, EC_{50}Ag10, EC_{50}Ag30) and their respective triplicates from setup 1. For visualization, rho ≧ 0.4 is shown in red dot, rho ≦ -0.4 is shown in green dot. The *p*-value is corrected using Benjamini-Hochberg correction (Benjamini and Hochberg, 1995), with $p < 0.05$ marked in red shade.

	Neg		LAg10		Pos		EC_{50}Ag10		EC_{50}Ag30	
	rho	*p*-value	rho	*p*-value	rho	*p*-value	rho	*p*-value	rho	*p*-value
G1	-0.5	0.07	-0.7	<0.05	-0.5	0.12	-0.8	<0.05	-0.6	<0.05
G2	-0.5	0.05	-0.4	0.13	-0.7	<0.05	-0.5	<0.05	-0.3	0.43
G3	0.2	0.55	0.4	0.20	-0.4	0.15	-0.7	<0.05	-0.7	<0.05
G4	-0.4	0.17	-0.5	0.06	-0.6	<0.05	0.5	0.06	0.4	0.13
G5	-0.4	0.21	0.3	0.32	-0.1	0.77	-0.4	0.19	-0.6	<0.05
G6	-0.6	<0.05	-0.5	0.11	-0.6	<0.05	-0.6	<0.05	-0.7	<0.05
G7	0.1	0.71	-0.5	0.10	-0.5	0.06	-0.7	<0.05	-0.3	0.27
G8	0.6	<0.05	0.7	<0.05	0.7	<0.05	-0.2	0.41	0.1	0.85
G9	0.5	0.05	0.4	0.12	0.6	<0.05	-0.4	0.14	0.2	0.57
G10	0.5	0.10	0.8	<0.05	0.6	<0.05	-0.3	0.33	-0.2	0.46
G11	-0.1	0.84	-0.6	<0.05	-0.5	0.06	0.6	<0.05	0.2	0.56
G12	0.5	0.05	-0.3	0.32	-0.1	0.76	0.6	<0.05	0.5	0.10
G13	-0.8	<0.05	-0.4	0.18	-0.6	<0.05	-0.1	0.69	-0.4	0.22
G14	-0.7	<0.05	-0.3	0.34	-0.7	<0.05	-0.7	<0.05	-0.6	<0.05
G15	-0.8	<0.05	-0.3	0.30	-0.8	<0.05	-0.5	0.06	-0.6	<0.05
G16	-0.7	<0.05	-0.3	0.35	-0.7	<0.05	-0.4	0.17	-0.3	0.41
G17	0.4	0.12	-0.1	0.86	-0.2	0.45	0.6	<0.05	0.5	0.05
G18	0.0	0.96	0.1	0.65	-0.2	0.54	-0.7	<0.05	-0.6	<0.05
G19	0.6	<0.05	0.3	0.26	-0.2	0.45	-0.6	<0.05	-0.4	0.19
G20	0.2	0.55	0.0	0.94	-0.4	0.13	-0.7	<0.05	-0.6	<0.05
G21	-0.8	<0.05	-0.7	<0.05	-0.6	<0.05	-0.8	<0.05	-0.8	<0.05
G22	-0.7	<0.05	-0.7	<0.05	-0.8	<0.05	0.1	0.86	-0.1	0.70
G23	-0.8	<0.05	-0.8	<0.05	-0.7	<0.05	0.3	0.35	0.0	0.95
G24	0.7	<0.05	0.5	0.05	0.6	<0.05	0.3	0.23	0.4	0.12
G25	-0.8	<0.05	-0.6	<0.05	-0.8	<0.05	-0.5	<0.05	-0.7	<0.05
G26	0.5	0.08	-0.1	0.69	-0.6	<0.05	0.0	0.99	0.2	0.58
G27	0.7	<0.05	0.7	<0.05	0.1	0.78	-0.1	0.81	0.2	0.51
G28	-0.7	<0.05	-0.7	<0.05	-0.7	<0.05	0.3	0.30	-0.1	0.84
G29	0.0	0.95	-0.2	0.57	-0.1	0.77	-0.7	<0.05	-0.4	0.25
G30	-0.4	0.18	0.0	0.91	-0.1	0.72	-0.6	<0.05	-0.6	<0.05
G31	0.7	<0.05	0.3	0.37	0.3	0.25	0.2	0.60	0.5	0.05
G32	0.7	<0.05	0.5	0.08	0.5	0.05	0.0	0.93	0.5	0.08
G33	0.6	<0.05	0.5	0.11	0.4	0.19	-0.3	0.27	0.2	0.63
G34	0.6	<0.05	0.4	0.15	0.0	0.89	-0.3	0.23	0.2	0.46

Figure S4. Cytometric diversity metrics (A, B, C and D) and biomass production (E). Data are shown for Setup 2 for Neg, LAg10, EC_{50}Ag10, EC_{50}Ag30, Pos. (A): Cytometric α-diversity values (threshold of 2.9 %), (B): Cytometric intra-community ß-diversity values (threshold 2.9 %), (C): Cytometric α-diversity values (a lower threshold of 0.71 %), (D): Cytometric intra-community ß-diversity values (a lower threshold of 0.71 %). Error bars are sample standard deviations from three parallel experiments. Comparable data for Setup 1 are shown in Figure 3.

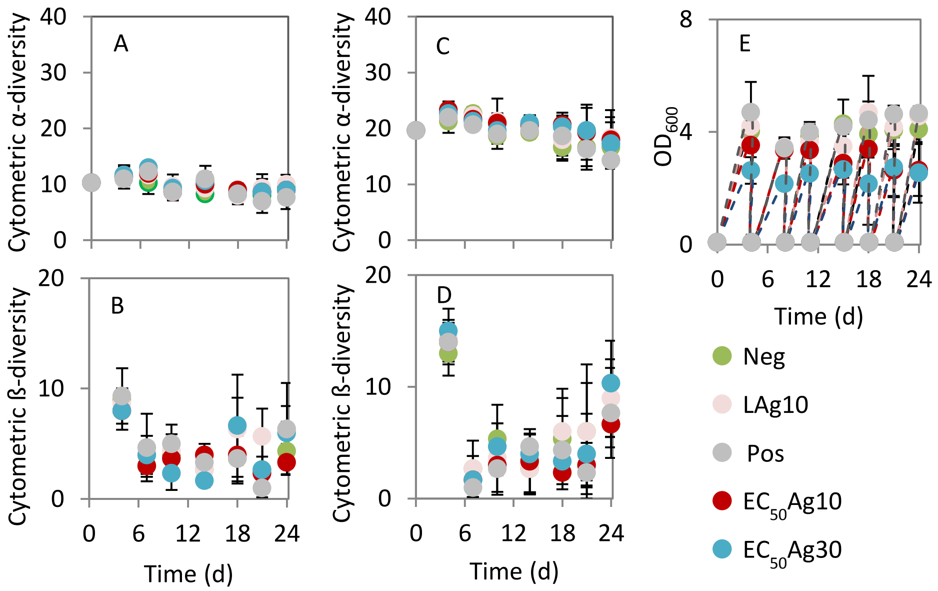

Sequencing workflow

DNA extraction and quality testing

DNA extraction: To extract a comparable amount of DNA from each fixed but not sorted control sample, cells were diluted in 70 µL PBS to a final optical density of 0.01 (d=5mm, ʎ=700nm). In parallel, by cell sorting, 500,000 cells were separated from each sample of interest. Following the protocol of Koch (Koch et al., 2013), the cells were pelleted by a centrifugation step (25 min, 20,000 g, 4 °C), respectively and the pellets were frozen at -20°C. After this freezing step, 70 µL of 10 % Chelex solution (Biorad, Hercules California, USA) was added to each pellet, followed by a heating step (45 min, 90 °C). A last centrifugation step (5min, 7,000 g, 4 °C) was needed to remove cell walls debris from the collected supernatant which contained the purified DNA (50 µL). The extracted DNA samples were stored at -20°C until library preparation.

DNA quality testing: The DNA extracted both from 500,000 sorted cells and the non-sorted communities was too low in quantity to be detected by the Qubit® 3.0 (Life Technologies, Carlsbad, California, USA) for quality control. Therefore, a PCR step was performed to evaluate the quality of the isolated DNA by testing their amplified products by gel-electrophoresis. The PCR step was done with 35 cycles in a S1000 Thermal cycler (Biorad) by using the universal primers Forward 27F 5'-AGAGTTTGATCMTGGCTCAG-3' and Reverse 1492R 5'-TACGGYTACCTTGTTACGACTT-3' following the recommendations of Lane (Lane et al., 1985). All tested DNA samples showed high quality results after amplification.

Mock strains and mock communities

Two mock species (*Rhodococcus sp.* RAH1 and *Pseudomonas putida* KT2440) and a mock community MBARC26 (Singer et al., 2016) were used as positive controls for the sequencing MiSeq run as well as positive controls for the sequencing data analysis pipeline. MBARC26, composed of 26 cultivable species (23 bacteria, 3 archaea) in different abundances was designed to mimic the diversity of a natural microbial community.

Library preparation for Illumina®

The V3-V4 region of the bacterial 16S rRNA gene region was the target of the used primers Pro341F 5'-CCTACGGGNBGCASCAG-3' (Takahashi et al., 2014) and Pro805R 5'-GACTACNVGGGTATCTAATCC-3' (Herlemann et al., 2011) synthesized by Eurofins (Eurofins Scientific, Luxembourg City, Luxembourg) as well as the 6-nt-barcoded primers for performing the library.

The PCR was performed in 10 µL containing 10 pmol of Forward and Reverse primers, 2 nmol of dNTP mix (Promega, Fitchburg, Wisconsin, USA), 2 µL 5x Phusion® GC solution, 20 nmol of $MgCl_2$, (both provided in the polymerase kit), 0.2 units of Phusion® High-Fidelity Polymerase (New England Biolabs,

Ipswich, Massachusetts, USA) and 1 µL DNA solution. Nuclease free water (Qiagen, Velno, Netherlands) was added to adjust the final reaction volume to 10 µL. The following PCR conditions were chosen: 3 min of initial denaturation at 95 °C, and a cycle of denaturation at 95 °C for 30 sec, annealing at 52 °C for 60 sec, extension for 1 min at 72 °C and a final extension at 72 °C for 10 min before storage at 4°C. The sorted samples were run for 20 PCR cycles and the non-sorted samples for 17. This difference is due to a lower DNA content in the sorted cells' DNA solution. For each reaction, a negative control without any DNA was amplified up to 35 cycles and checked via gel electrophoresis (1.5 % agarose) to ensure that no contamination was present. In absence of contamination, the procedure continued by the purification of the amplicons by using the Agencourt® AMPure® XP-Kit (Beckman Coulter, Brea, California, USA) following their recommended protocol. The purified DNA fragments were resuspended in nuclease free water (Qiagen, Velno, Netherlands) up to 10 µL. The second step PCR took place within the same conditions as mentioned above by using 1 µL of the purified amplicon solution and the sample-corresponded barcoded primers for 8 cycles. Again, negative controls were run in parallel without DNA up to 35 PCR cycles to test potential contaminations. Samples free of contamination were purified as described before; this DNA was quantified by using the Qubit® 3.0 (Life technologies, Carlsbad, Califorania, USA) and the HS DNA kit (Life technologies, Carlsbad, California, USA), to finally be equimolarly pooled and be sequenced on a MiSeq sequencing machine (Illumina, San Diego, California, USA) at the Helmholtz Center for Infection Research Braunschweig by the Genome Analytics Group (GMAK, HZI, Braunschweig, Germany). To minimize the technical bias, every PCR was done in triplicates, purified and quantified separately before to be pooled as a unique sample. The mock communities were also processed in the same way.

Sequencing data evaluation procedure

The Illumina dataset was firstly quality trimmed with PRINTSEQ (Schmieder and Edwards, 2011) from the 3' side at a minimum of Q=30 in a window of 20 bases. The remaining sequences were demultiplexed and merged by using Mothur version 1.39 (Schloss et al., 2009). The pre-clustering step helped us to remove the singletons from the data set and the chimeras were removed by using UCHIME (Edgar et al., 2011). The OTU classification was done by using the Mothur's average neighbour clustering algorithm with a 97 % sequence similarity cut off on the SILVA database version 128 (Quast et al., 2013). The obtained data sets comprised between 4,483 cleaned reads for the sample 24_Ag30 which was kept as subsampling threshold for the normalization procedure (to allow comparison of diversity between samples) and 56,622 cleaned reads obtained for the sample 7_Pos G3. All the raw data are available under the BioProject accession number: PRJNA400127.

OTU threshold determination: Our sequencing analysis allowed us to recover the two mock species and 23 species out of the 26 composing the MBARC26 mock community. The three missing strains (*N. dassonvillei, S. rotundus, and S. enterica*) were probably lost due to their low abundance level and the sequencing technique we used for the metaprofiling (MiSeq, V3 kit, 2 x 300 bp (V3 kit, Illumina, USA)) which was different from the Singer's *et al.* workflow (Singer et al., 2016) conducted on a higher resolving metagenomic approach by using PacBio and HiSeq. Nonetheless, the data of the mock strains and mock community confirmed the reliability of our workflow. The raw data are available under the BioProject accession number: PRJNA387753, Biosamples: SAMN07187887, SAMN07187792, SAMN07187791. In parallel we used the MBARC26 sequencing data to set the confidant OTU threshold for cleaned reads to 0.71 % as was recommended by Bokulich *et al.* (Bokulich et al., 2012) meaning that species with abundance below this 0.71 % OTU threshold were not included in the data evaluation. Nonetheless, this threshold allowed us to study the most abundant OTUs in our samples and probably the key species involved in the microbiological process.

The gamma diversity we obtained from these 21 samples, represented by 94,143 forward-reverse overlapped subsampled sequences, comprised 32 different OTUs at an OTU threshold of 0.71 %. Instead, the raw data provide 2,175 OTUs, hinting at a huge diversity in the rare biosphere of our samples.

Figure S5. Rarefaction curves of 21 raw data sets of sorted and unsorted samples (from Setup 1) with a maximum diversity of 1,626 OTUs for the sample inoculum. The green line marks the lowest cleaned data set composed 4,483 reads for the sample 24_EC_{50}Ag30_3.

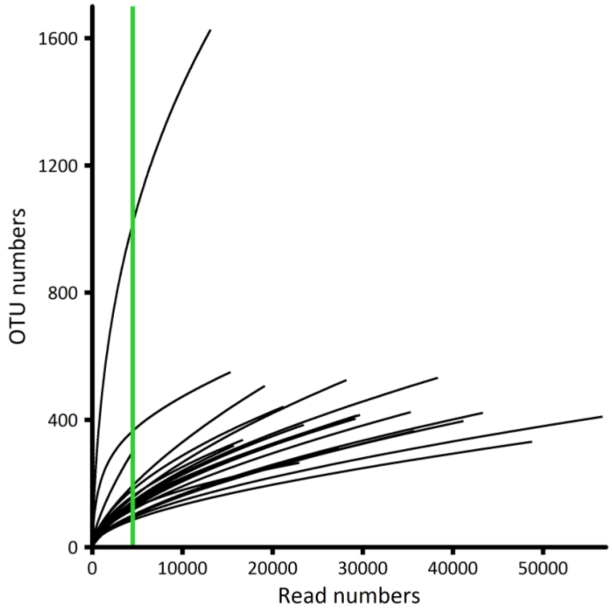

Figure S6. Rarefaction curves of the 21 samples sorted and unsorted (from Setup 1) at an OTU threshold of 0.71 %. The green line marks the subsampling cleaned read number at 4,483. Due to the OTU threshold the black curves show the most abundant OTU numbers per sample. Information about OTUs below the mentioned threshold is not part of the analysis.

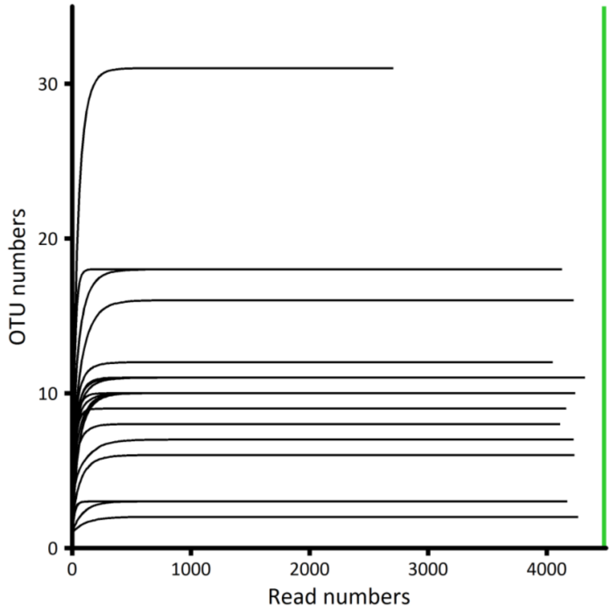

Table S5. Detail information of Figure 4 on class and relative genus level with cell number abundance per time point (7 d, 24 d), treatment (Neg, LAg10, Pos, EC_{50}Ag10, EC_{50}Ag30), one of the triplicates (1, 2, or 3). Maximum abundance is 1.00. Same colors are used for each class as in Figure 4.

Class	Genus per class	Genus abundance per class (whole community samples)							
		Inoculum_2	7_Neg_1	7_LAg10_2	24_Neg_1	24_LAg10_2	24_Pos_3	24_EC_{50}Ag10_3	24_EC_{50}Ag30_3
Bacilli	*Lactococcus*		1.00	1.00	0.77	0.57	1.00	0.83	0.44
	Vagococcus				0.23	0.43		0.17	0.56
Flavobacteria	*Empedobacter*		0.74	0.67			1.00		0.05
	Myroides	1.00	0.26	0.29	1.00	1.00			0.95
	Flavobacterium			0.04					
Alphaproteobacteria	*Brevundimonas*		0.70	0.49	0.69	0.50	0.67	1.00	0.28
	Paenochrobactrum		0.18	0.13	0.31	0.21	0.20		0.72
	Pseudochrobactrum		0.11	0.38		0.29	0.13		
	Rhodobacteraceae_unclassified	1.00							
Betaproteobacteria	*Alcaligenes*		0.10	0.48	1.00	1.00	1.00		
	Comamonas		0.90	0.52					
	Leptothrix	1.00							
	Bordetella								1.00
Gammaproteobacteria	*Acinetobacter*		0.74	0.75	0.23			0.36	
	Enterobacteriaceae_unclassified		0.11	0.25				0.03	
	Providencia		0.08		0.77	1.00			
	Escherichia-Shigella		0.07					0.56	0.07
	Candidatus_Competibacter	0.67							
	Dokdonella	0.33							
	Morganella						0.80	0.02	0.64
	Stenotrophomonas						0.05		0.29
	Proteus						0.07		
	Serratia						0.08		
	Haemophilus							0.02	
Sphingobacteriia	*Sphingobacterium*		1.00	1.00					
	Terrimonas	0.55							
	Saprospiraceae_unclassified	0.45							
Saccharibacteria	*Saccharibacteria_unclassified*	1.00		1.00					
Nitrospira	*Nitrospira*	1.00							
Ignavibacteria	PHOS-HE36_unclassified	1.00							
Acidimicrobiia	CL500-29_marine_group	1.00							
Blastocatellia	*Stenotrophobacter*	1.00							
Cytophagia	*Chryseolinea*	1.00							
Chlorobi	SJA-28_unclassified	1.00							
Clostridia	*Syntrophobotulus*							0.43	
	D8A-2_unclassified							0.57	
Bacteroidia	*Dysgonomonas*								1.00

Class	Genus per class	Genus abundance per class (cell increasing gates)			
		24_Neg_3_G4	24_Neg_3_G11	24_EC_{50}Ag30_2_G4	24_EC_{50}Ag30_2_G11
Bacilli	*Lactococcus*	1.00	1.00	0.99	0.99
	Aminobacterium			0.01	
	Neisseria				0.01
Flavobacteria	*Empedobacter*	1.00	0.87		
	Myroides		0.13		
Alphaproteobacteria	*Brevundimonas*	0.32	0.34		
	Paenochrobactrum	0.20	0.35		
	Pseudochrobactrum	0.48	0.31		
Betaproteobacteria	*Alcaligenes*	0.30	0.73		
	Comamonas	0.70	0.27		
Gammaproteobacteria	*Enterobacteriaceae_unclassified*	1.00	0.93		
	Morganella		0.07		
Sphingobacteriia	*Sphingobacterium*	1.00	1.00		

Class	Genus per class	Genus abundance per class (cell decreasing gates)	
		7_EC_{50}Ag10_1_G3	7_EC_{50}Ag30_1_G3
Flavobacteria	*Empedobacter*	0.33	0.96
	Myroides	0.66	
	Flavobacterium	0.01	0.01
	Elizabethkingia		0.02
	Chryseobacterium		0.01
Alphaproteobacteria	*Paenochrobactrum*	0.82	0.77
	Pseudochrobactrum	0.18	0.23
Gammaproteobacteria	*Acinetobacter*	1.00	
Sphingobacteriia	*Sphingobacterium*	1.00	1.00
Bacteroidia	*Dysgonomonas*		1.00

References

Benjamini, Y., and Hochberg, Y. (1995). Controlling the false discovery rate: a practical and powerful approach to multiple testing. *J. R. Stat. Soc.* 57, 289–300.

Bokulich, N. A., Subramanian, S., Faith, J. J., Gevers, D., Gordon, J. I., Knight, R., et al. (2012). Quality-filtering vastly improves diversity estimates from Illumina amplicon sequencing. *Nat. Methods* 10, 57–59. doi:10.1038/nmeth.2276.

Edgar, R. C., Haas, B. J., Clemente, J. C., Quince, C., and Knight, R. (2011). UCHIME improves sensitivity and speed of chimera detection. *Bioinformatics* 27, 2194–2200. doi:10.1093/bioinformatics/btr381.

Herlemann, D. P., Labrenz, M., Jürgens, K., Bertilsson, S., Waniek, J. J., and Andersson, A. F. (2011). Transitions in bacterial communities along the 2000 km salinity gradient of the Baltic Sea. *ISME J.* 5, 1571–1579. doi:10.1038/ismej.2011.41.

Koch, C., Günther, S., Desta, A. F., Hübschmann, T., and Müller, S. (2013). Cytometric fingerprinting for analyzing microbial intracommunity structure variation and identifying subcommunity function. *Nat. Protoc.* 8, 190–202. doi:10.1038/nprot.2012.149.

Lane, D. J., Pace, B., Olsen, G. J., Stahl, D. A., Sogin, M. L., and Pace, N. R. (1985). Rapid determination of 16S ribosomal RNA sequences for phylogenetic analyses. *Proc. Natl. Acad. Sci. U. S. A.* 82, 6955–6959.

Quast, C., Pruesse, E., Yilmaz, P., Gerken, J., Schweer, T., Yarza, P., et al. (2013). The SILVA ribosomal RNA gene database project: improved data processing and web-based tools. *Nucleic Acids Res.* 41, D590–D596. doi:10.1093/nar/gks1219.

Schloss, P. D., Westcott, S. L., Ryabin, T., Hall, J. R., Hartmann, M., Hollister, E. B., et al. (2009). Introducing mothur: open-source, platform-independent, community-supported software for describing and comparing microbial communities. *Appl. Environ. Microbiol.* 75, 7537–7541. doi:10.1128/AEM.01541-09.

Schmieder, R., and Edwards, R. (2011). Quality control and preprocessing of metagenomic datasets. *Bioinformatics* 27, 863–864. doi:10.1093/bioinformatics/btr026.

Singer, E., Andreopoulos, B., Bowers, R. M., Lee, J., Deshpande, S., Chiniquy, J., et al. (2016). Next generation sequencing data of a defined microbial mock community. *Sci. Data* 3, 160081. doi:10.1038/sdata.2016.81.

Takahashi, S., Tomita, J., Nishioka, K., Hisada, T., and Nishijima, M. (2014). Development of a prokaryotic universal primer for simultaneous analysis of *Bacteria* and *Archaea* ssing next-generation sequencing. *PLoS ONE* 9, e105592. doi:10.1371/journal.pone.0105592.
